# Multiple domains of bacterial and human Lon proteases define substrate selectivity

**DOI:** 10.1038/s41426-018-0148-4

**Published:** 2018-08-17

**Authors:** Lihong He, Dongyang Luo, Fan Yang, Chunhao Li, Xuegong Zhang, Haiteng Deng, Jing-Ren Zhang

**Affiliations:** 1 Center for Infectious Disease Research, School of Medicine, Tsinghua University, Beijing, China; 2 MOE Key Laboratory of Bioinformatics, Bioinformatics Division, TNLIST and Department of Automation, Tsinghua University, Beijing, China; 3 MOE Key Laboratory of Bioinformatics, School of Life Sciences, Tsinghua University, Beijing, 10084 China; 4 Philip Research Institute for Oral Health, School of Dentistry, Virginia Commonwealth University, Richmond, VA USA; 5 Collaborative Innovation Center for Biotherapy, State Key Laboratory of Biotherapy and Cancer Center, West China Hospital, West China Medical School, Sichuan University, Chengdu, China

## Abstract

The Lon protease selectively degrades abnormal proteins or certain normal proteins in response to environmental and cellular conditions in many prokaryotic and eukaryotic organisms. However, the mechanism(s) behind the substrate selection of normal proteins remains largely unknown. In this study, we identified 10 new substrates of *F. tularensis* Lon from a total of 21 candidate substrates identified in our previous work, the largest number of novel Lon substrates from a single study. Cross-species degradation of these and other known Lon substrates revealed that human Lon is unable to degrade many bacterial Lon substrates, suggestive of a “organism-adapted” substrate selection mechanism for the natural Lon variants. However, individually replacing the N, A, and P domains of human Lon with the counterparts of bacterial Lon did not enable the human protease to degrade the same bacterial Lon substrates. This result showed that the “organism-adapted” substrate selection depends on multiple domains of the Lon proteases. Further in vitro proteolysis and mass spectrometry analysis revealed a similar substrate cleavage pattern between the bacterial and human Lon variants, which was exemplified by predominant representation of leucine, alanine, and other hydrophobic amino acids at the P(−1) site within the substrates. These observations suggest that the Lon proteases select their substrates at least in part by fine structural matching with the proteins in the same organisms.

## Introduction

Lon is a member of the AAA+ (ATPases associated with various cellular activities) protease superfamily with a wide distribution in bacteria, archaea, and eukaryotes^1^. In bacteria, Lon contributes to proteolytical regulation of many important functions, including encapsulation^2^, genetic competence^3^, motility^4^, heat-shock response^5^, persister formation^6^ and drug resistance^7^, DNA replication and repair^8,9^, and production of virulence factor^10^. Our previous study suggests that *Francisella tularensis* Lon is a heat-shock protease with the observation that its transcript level was increased 2.5-fold by heat stress^11^; and its promoter contains a putative RpoH-binding site^12^. Moreover, *F. tularensis* Lon is required for bacterial stress tolerance and infection of mammalian hosts^11^. Since human Lon (hLon) or LONP1 is localized to the mitochondrial matrix^13^, the current knowledge on LONP1 centers on its role in the subcellular environment. LONP1 is involved in the control of mitochondrial matrix protein quality^14^, maintenance of mitochondrial DNA nucleoid integrity^15^, response to hypoxia and oxidative stress^16,17^, and regulation of mitochondrial metabolism^18^. LONP1 dysfunction has been implicated in aging^19^, cancer, and CODAS syndrome^20^.

The active Lon protease forms a hexameric ring-shaped structure with a central pore, consisting of a substrate unfolding chamber and a proteolysis chamber^21,22^. Based on the domain architecture, Lon is divided into two subfamilies: LonA (in bacteria and eukaryotes) and LonB (in archaea). LonA is composed of three functional domains: an amino (N)-terminal domain (N domain) responsible for oligomerization and interactions with the substrate^23^; a central ATPase domain (A domain) required for ATP binding and hydrolysis; and a carboxyl (C)-terminal proteolytic domain (P domain) for substrate degradation^1^. As a member of the LonA family, the human LONP1 has three different isoforms generated by alternative splicing. The longest isoform (959 amino acids) is regarded as the canonical form, which possesses an extra mitochondria-targeting sequence of 114 amino acids at the N terminus^24^. The functions of isoforms 2 (missing amino acids 42–105 of isoform 1) and 3 (missing amino acids 1–196) are unknown although the truncations in both the isoforms may affect their mitochondrial localization. While the precise contribution of the three Lon domains to substrate selection is unknown, the N domain of the *Escherichia coli* Lon (eLon) is required for substrate recognition and binding as exemplified by the direct binding between Lon and sul20 peptide^23^. Consistently, a E240K mutation in the N domain selectively alters degradation of substrates^25^. Furthermore, deletion of 124–304 amino acids in the N domain led to a complete loss of the proteolytic activity of eLon toward its substrate β-casein^26^. A recent report revealed that LONP1 mutant with a Y565H mutation in the A domain could not bind or degrade its substrates^27^.

Bacterial Lon is regarded as a major protease for degradation of misfolded proteins. This is exemplified by the observation that approximately 50% of the misfolded proteins in *E. coli* are degraded by Lon^28^. However, certain proteins are still subjected to Lon degradation even under their native conditions, such as HUβ^29^, IbpA^5^, RcsA^30^, RpsB^31^, SoxS^32^, and SulA^33^ in *E. coli*. While it is largely unknown how Lon selects normal proteins for degradation, several sequence features on the known substrates contribute to their degradation by Lon. Certain proteins are degraded by Lon on the basis of short substrate sequences, referred to as degradation tags or degrons^34^. Previous studies identified degrons in several substrates, including residues 49–68 of β-galactosidase (β20)^35^, residues 15–29 of UmuD^36^, the C-terminal 20 residues of SulA (sul20)^37^, and the N-terminal 21 residues of SoxS^32^. Attaching a degradation tag to a stably folded protein renders them degradable by Lon^35^. A special degron “SsrA tag” also contributes to substrate degradation by Lon^38^. In *E. coli*, the “SsrA tag” sequence (AANDENYALAA) was appended to the C terminus of proteins when incomplete translation occurs^39^. ClpXP is responsible for >90% of the degradation of SsrA-tagged proteins, whereas Lon contributes about 2% to the degradation^40^. Furthermore, degradation of certain substrates also requires adaptors. Recently, *Bacillus subtilis* SmiA was shown to facilitate degradation of SwrA by Lon^4^. Furthermore, *Yersinia pestis* HspQ acts as a Lon specificity-enhancing factor and enhances Lon-mediated degradation of a select set of substrates (e.g. YmoA, RsuA, Y0390, and Fur)^41^. In contrast, the bacteriophage T4 PinA protein prevents the degradation of some Lon substrates (e.g. Casein and CcdA)^42^. A recent study revealed that degradation of some DNA-binding substrates (e.g. TrfA and RepE) requires Lon interaction with DNA^43^.

There is a limited number of Lon substrates identified in both prokaryotic and eukaryotic organisms. Our preliminary search of literature identified 12, 3, and 9 substrates in *E. coli*, *B. subtilis*, and human, respectively. The 5 substrates of *F. tularensis* Lon described in our previous work represent the largest number of the Lon substrates identified in a single study^11^. In this work, we tested a set of 21 *F. tularensis* Lon substrate candidates identified in our previous study^11^, and verified 10 proteins as authentic substrates by in vivo proteolysis. These and other known Lon substrates were used to determine cross-species degradation by bacterial and human Lon proteases. These experiments showed that hLon failed to degrade many *F. tularensis* and eLon substrates, indicative of “organism-adapted” substrate specificity among the natural Lon variants. The potential mechanisms of this organism adaptation were further investigated by domain swap between bacterial and human Lon variants.

## Results

### Identification of novel Lon substrates

**Fig. 1:**
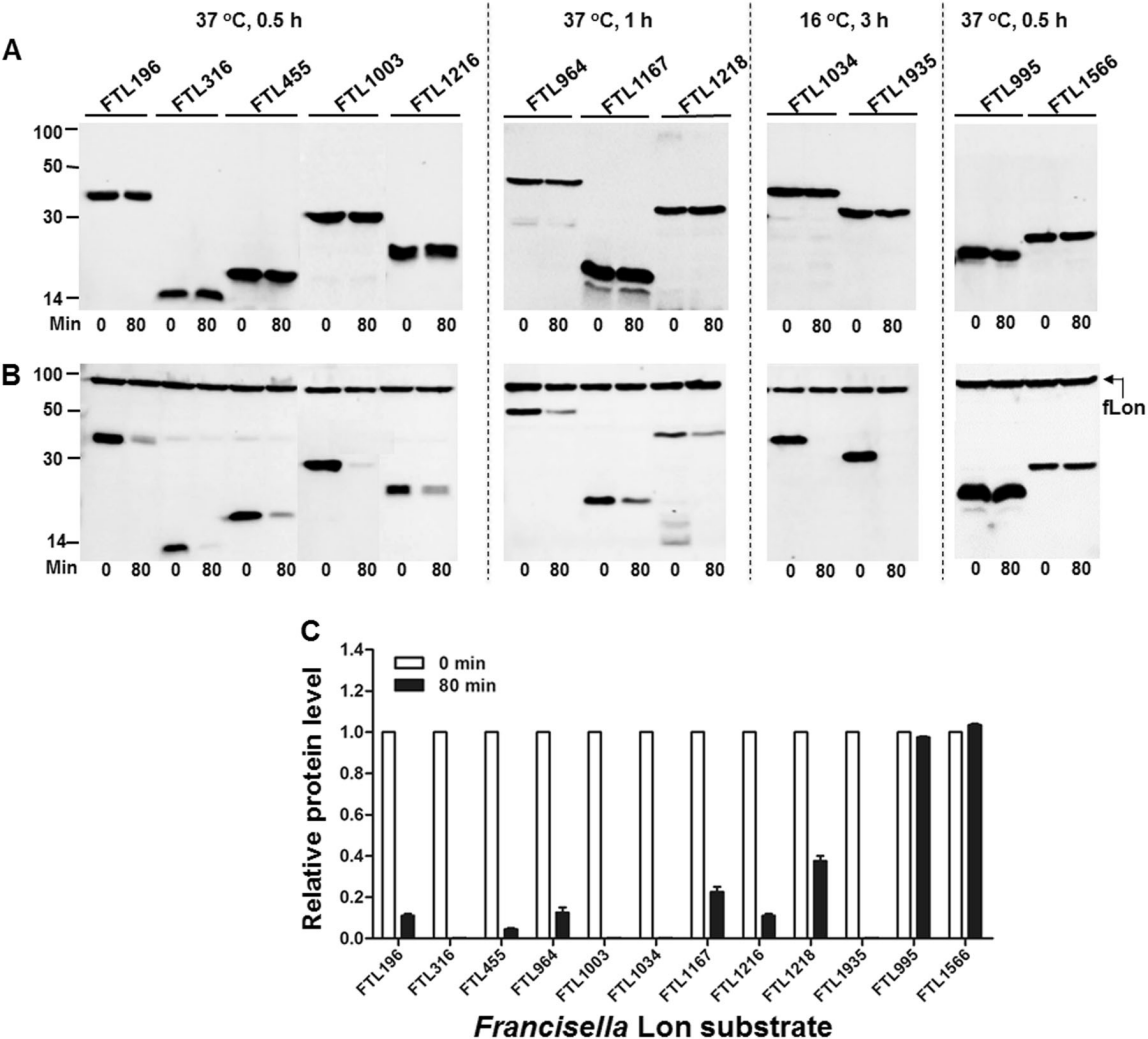
Degradation of the *F. tularensis* proteins by fLon in *E. coli* ER2566 (Lon^−^). Stability of the *F. tularensis* proteins in the absence (**a**) or presence (**b**) of the fLon-expression plasmid in Lon-deficient *E. coli* ER2566. fLon was induced with arabinose for 2 h before induction of the target proteins with IPTG under the conditions specified at the top of each panel, and subsequently treatment with spectinomycin. The cells were harvested at 0 and 80 min after the addition of spectinomycin; each target protein detected by immunoblotting using the anti-His_6_ antibody. The sizes of protein standards are indicated at the left side in kDa. **c** The amount of each target protein in **b** was quantified by Image Lab. The level of each protein at 80 min is presented as a value relative to that at 0 min. Bars represent the mean value ± SEM (*n* = 3)

Our previous proteomic approach identified 5 Lon substrates from a total of 29 putative substrates in *F. tularensis*, which were significantly enriched in the absence of the Lon protease^11^. We sought to validate degradation of the remaining 24 putative proteins by in vivo proteolysis (Table S3). Protein degradation was initially assessed by expressing the coding DNA sequences of the *Francisella* proteins with an isopropyl-β-d-1-thiogalactopyranoside (IPTG)-inducible promoter in *E. coli*. Proteolytic degradation of the target proteins was evaluated by monitoring protein stability in the presence or absence of the *Francisella* Lon (fLon) that was driven by an arabinose-inducible promoter. In all, 21 out of the 24 proteins were successfully expressed in soluble form and remained stable in the absence of the fLon protease (Fig. 1a; not shown). However, co-production of the fLon made 10 proteins undetectable (FTL316, FTL1003, FTL1034, and FTL1935) or substantially reduced (FTL196, FTL455, FTL964, FTL1167, FTL1216, and FTL1218) within 80 min (Fig. 1b), strongly suggesting that these proteins are authentic substrates of the fLon. In sharp contrast, the other 11 *Francisella* proteins, as exemplified by FTL995 and FTL1566, remained relatively stable upon exposure to the fLon (Fig. 1b, Table S3, and not shown). The result suggested that these 11 *Francisella* proteins are not substrates of Lon under these conditions.

**Fig. 2:**
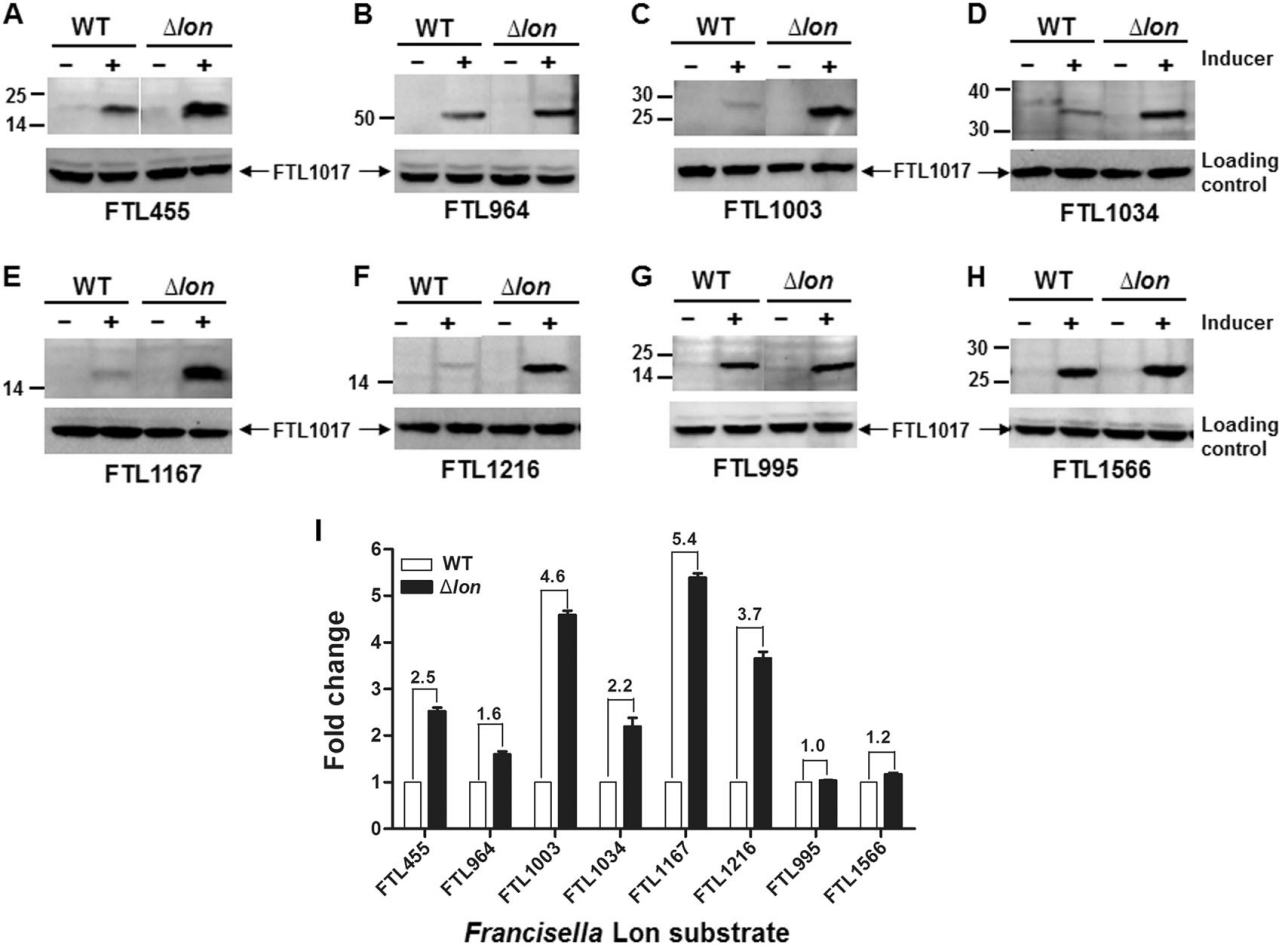
Stability of the *Francisella* Lon substrates in the LVS and *∆lon* strains. Each target gene in the shuttle plasmid pEDL17 was expressed with a His tag from a tetracycline-inducible promoter in LVS (open bar) or isogenic *∆lon* mutant (filled bar). Proteins were detected by immunoblotting (**a**–**h**) and quantified by Image Lab (**i**) as in Fig. 1. Abundance ratio of each protein between LVS and the *∆lon* mutant is indicated at the top of relevant bars. Each bars represents the mean value ± SEM (*n* = 3). The protein encoded by endogenous (chromosomal) FTL1017 was detected with an antiserum as a loading control

We further validated Lon degradation of the 10 proteins in *F. tularensis* live vaccine strain (LVS) as described in our previous study^11^. The pEDL17 derivatives containing their coding sequences were transformed into LVS or isogenic ∆*lon* mutant to express C-terminally His-tagged proteins in a tetracycline-inducible manner. Specific degradation by fLon was quantified by immunoblotting comparison of the protein abundance between the parent and ∆*lon* strains. Upon anhydrotetracycline (ATc) induction, FTL455 (Fig. 2a), FTL964 (Fig. 2b), FTL1003 (Fig. 2c), FTL1034 (Fig. 2d), FTL1167 (Fig. 2e), and FTL1216 (Fig. 2f) were successfully expressed in both strains, but appeared to be more abundant in the ∆*lon* strain (Fig. 2a–f). The remaining four proteins (FTL196, FTL316, FTL1218, and FTL1935) were undetectable (not shown) under the same conditions. As non-substrate controls, the protein levels of FTL995 (Fig. 2g) and FTL1566 (Fig. 2h) were similar in both LVS and the ∆*lon* strains after the induction. Quantification of the immunoblotting signals revealed that all of the six proteins were more abundantly present in the ∆*lon* mutant (Fig. 2i). These results thus validated that FTL455, FTL964, FTL1003, FTL1034, FTL1167, and FTL1216 are substrates of fLon in the native host bacteria.

To identify any features shared by the Lon substrates, we compared the 15 Lon candidates identified in this and our previous study^11^, in terms of their sequence similarity, cellular localization, and secondary structure. All of these proteins encode no signal peptide sequence as predicted by SignalP 4.1 (http://www.cbs.dtu.dk/services/SignalP/)^44^; no transmembrane region as predicted by TMHMM 2.0 (http://www.cbs.dtu.dk/services/TMHMM/)^45^, indicating that they appear to be localized in cytoplasm. Seven of these proteins contain <200 amino acids, suggesting that Lon prefers relatively small proteins (Table S4). Except for a high similarity between FTL663 and FTL1217 (45.2% amino-acid identity), there are no obvious sequence features shared by the 15 proteins. Consistent with the previous observation that Lon predominantly cleaves substrates at the hydrophobic amino acids^29,37^, 8 of the 15 Lon substrates were composed of more than 40% nonpolar amino acids (FTL196, FTL578, FTL964, FTL1003, FTL1167, FTL1216, FTL1228, and FTL1935) (Table S4). However, this trial did not reveal any obvious biochemical characteristics shared by all of the Lon substrates although they represent an extreme small fraction of the 1800 cellular proteins encoded by the *Francisella* genomes^46–48^.

In summary, our in vivo proteolysis screen led to the identification of 10 new Lon substrates from a total of 21 soluble candidate proteins. This high success rate (47.6%) demonstrates the reliability of our approach in identification of Lon substrates. Because Lon degrades only small number of proteins encoded by the *Francisella* proteome, this result also shows that Lon strictly selects its substrates as compared with other serine proteases with promiscuous substrate selectivity, such as trypsin and subtilisin^1,49^.

### Unique substrate specificity of the Lon variants

**Fig. 3:**
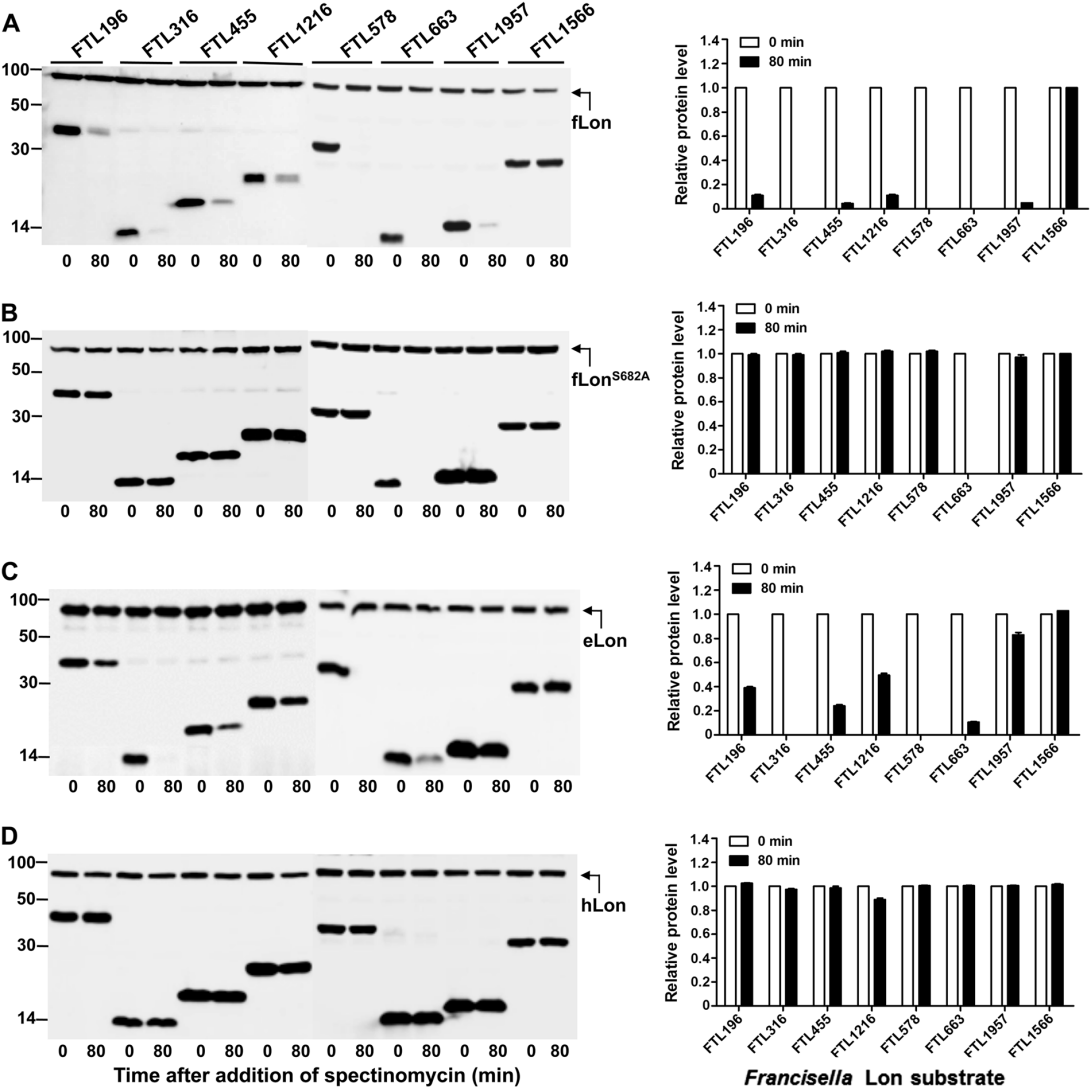
Cross-species degradation of the fLon substrates by the Lon variants of *E. coli* and human Lon variants. Stability of the fLon substrates was detected in the presence of fLon (**a**), fLon^S682A^ (**b**), eLon (**c**), or hLon (**d**) in *E. coli* ER2566. Each set of the *lon* and target genes were cloned in two compatible plasmids behind either arabinose (for Lon)- or IPTG (for substrate)-inducible promoter. Protein detection and quantification were carried out as in Fig. 1

In the context of strict substrate selectivity by fLon, we wondered whether the fLon substrates are degradable by its natural variants from other species (i.e. *E. coli* and human). Sequence comparison revealed that fLon shares 53.7% and 40.4% of amino-acid sequence identity with eLon and hLon, respectively. The stability of seven fLon substrates was tested in the presence of the Lon proteases from *E. coli* and human (Fig. 3). These proteins were selected because they were abundantly expressed upon induction in *E. coli*. As revealed earlier (Fig. 1a), all of the seven proteins were undetectable (FTL316, FTL578, and FTL663) or diminished (FTL196, FTL455, FTL1216, and FTL1957) in the presence of fLon (Fig. 3a). These proteins except for FTL663 were stable in the presence of proteolytically inactive fLon^S682A^ (Fig. 3b), thus confirming that they are fLon substrates. The instability of FTL663 was independent of the protease activity of fLon, indicating that fLon may promote degradation of FTL663 by other proteases. eLon degraded two proteins (FTL316 and FTL578) as efficiently as fLon, but showed obviously weaker activity with the other five proteins (FTL196, FTL455, FTL1216, FTL1663, and FTL1957) (Fig. 3c). In the presence of eLon, FTL316 and FTL578 became undetectable, whereas four other proteins were substantially diminished; the abundance of FTL1957 was only marginally reduced (by 15%). Surprisingly, none of the seven fLon substrates was degraded by hLon (Fig. 3d), although the expression of the hLon construct led to degradation of the known hLon substrates under the same conditions (see below). These results revealed species-specific substrate selectivity of the Lon variants.

**Fig. 4:**
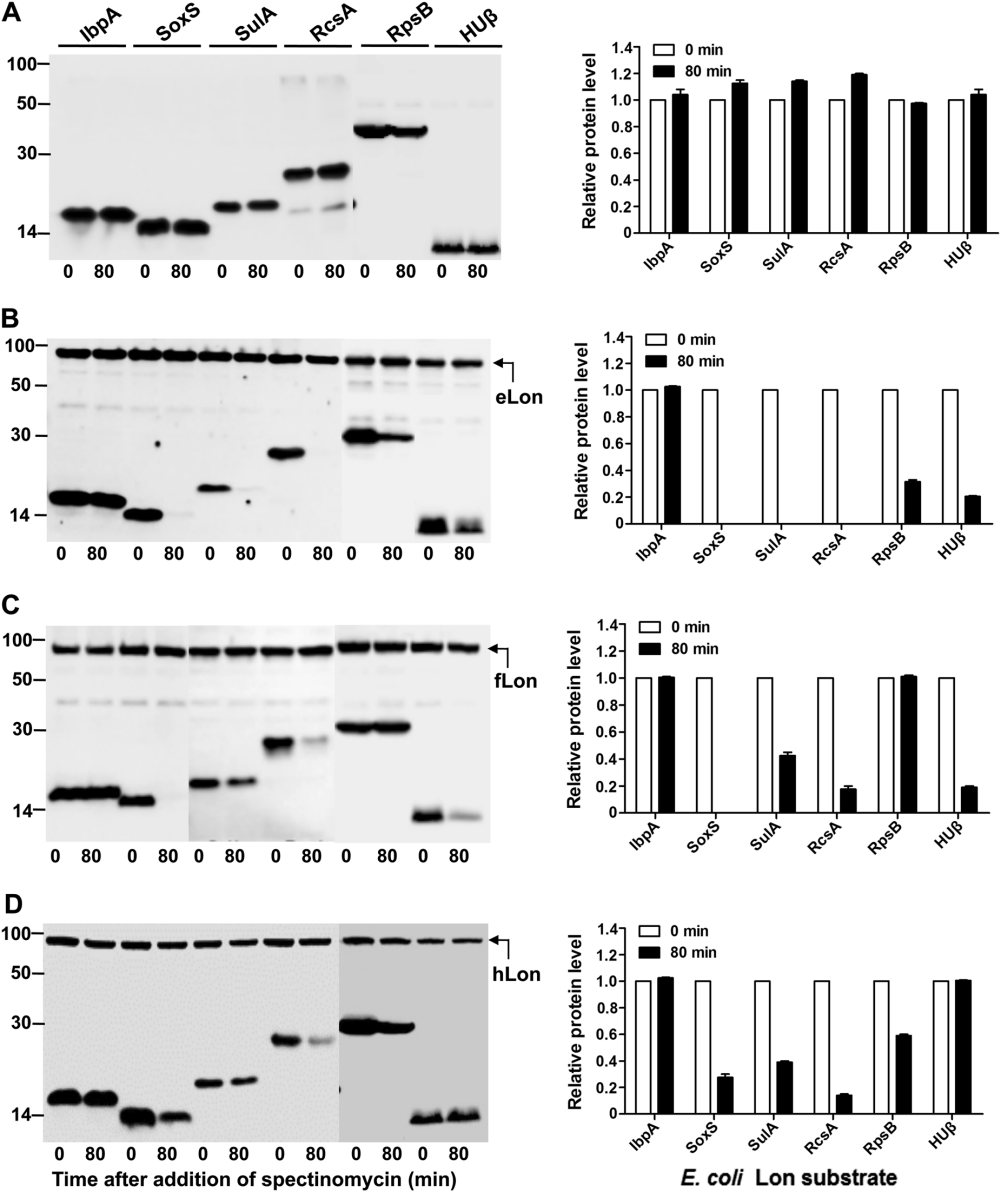
Cross-species degradation of the eLon substrates by *Francisella* and human Lon proteases. Stability of the eLon substrates was detected in the absence (**a**) or presence of eLon (**b**), fLon (**c**), or hLon (**d**) as in Fig. 3

We further tested substrate selectivity of the Lon variants with the known eLon substrates. Six *E. coli* proteins (IbpA, SoxS, SulA, RcsA, RpsB, and HUβ) were selected on the basis of previous studies^5,29–31,50,51^. All of these proteins were stable in the absence of the inducer (Fig. 4a) and in the presence of eLon^S679A^ (Fig. S1A), but became undetectable (SoxS, SulA, and RcsA) or substantially reduced (RpsB and HUβ) except for IbpA in the presence of eLon (Fig. 4b). IbpA, a small heat-shock protein, was previously identified as a eLon substrate^5^, but its abundance was not affected by co-expression with eLon (Fig. 4b, lanes 1 and 2). Bissonnette et al.^5^ reported that purified ^35^S-labeled IbpA could be degraded by eLon. This discrepancy could be due to a higher detection sensitivity of the radioactive method used in the previous study. When tested with the variants of fLon and hLon, three of the eLon substrates (SoxS, SulA, and RcsA) were also degraded by fLon (Fig. 4c) and hLon (Fig. 4d). HUβ was degraded by fLon but remained stable in the presence of hLon. In a similar manner, RpsB was diminished after the expression of hLon, but its level did not significantly change in the presence of fLon. These results provide additional evidence that natural variants of the Lon proteases possess unique substrate specificity.

**Fig. 5:**
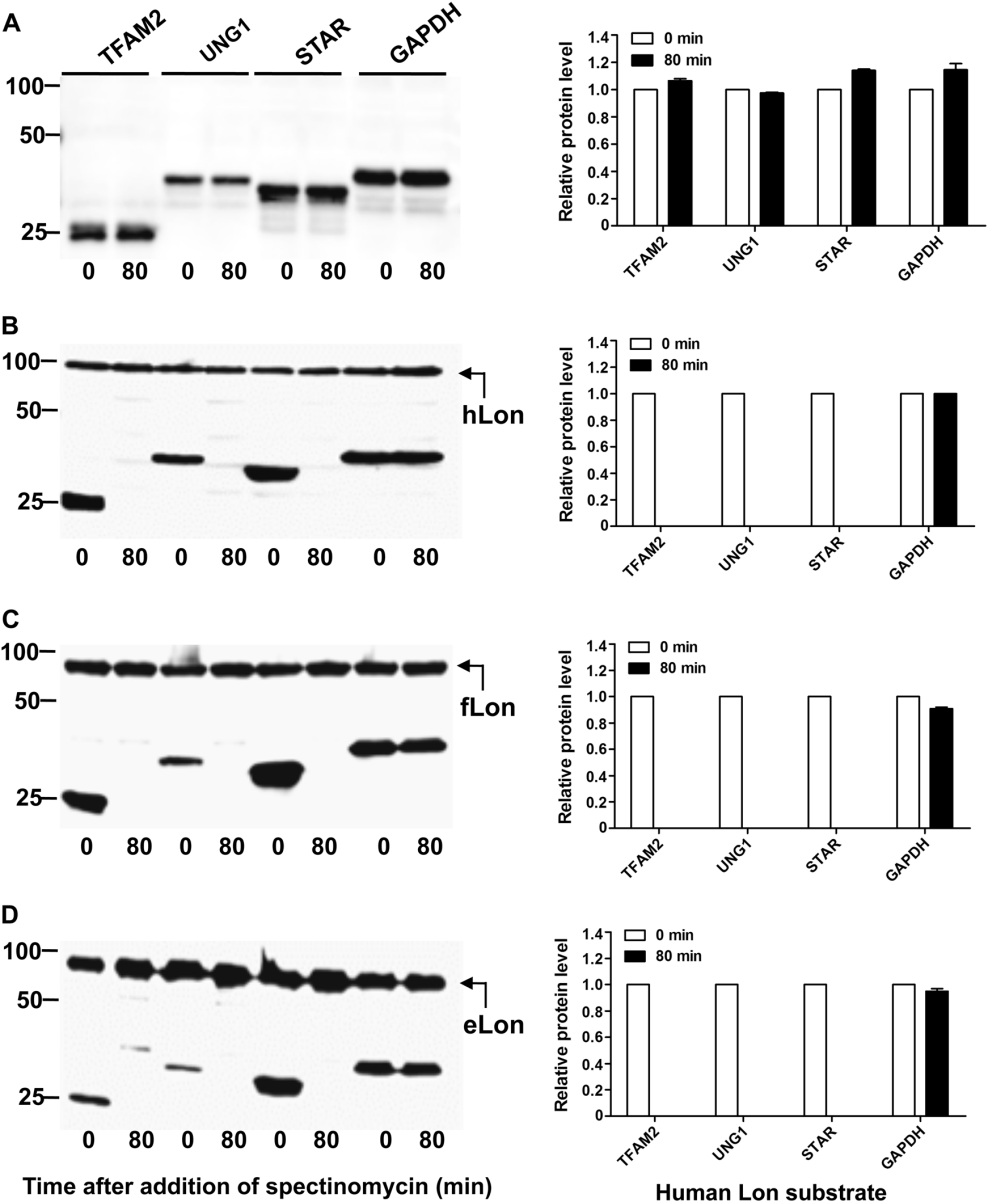
Cross-species degradation of the hLon substrates by bacterial Lon proteases. Stability of the human Lon substrates was detected in the absence (**a**) or presence of hLon (**b**), fLon (**c**), or eLon (**d**) as in Fig. 3

Lastly, we tested proteolysis of the known hLon substrates by the three Lon variants. Among five human proteins tested, TFAM2, UNG1, and STAR were adequately expressed in *E. coli* after codon optimization of the coding sequences (Fig. 5a). These proteins are described as the substrate of hLon^20^. Consistent with the literature, TFAM2, UNG1, and STAR were highly susceptible to proteolysis by hLon since they were undetectable once the expression of hLon was induced (Fig. 5b). Consistently, all of the three proteins were relatively stable in the presence of enzymatically inactive hLon^S855A^ (Fig. S1B). When tested with bacterial Lon variants, all of the three human proteins were effectively degraded by both fLon (Fig. 5c) and eLon (Fig. 5d). As a non-substrate control, the abundance of GAPDH remained relatively constant in the presence of hLon. Together, these results have demonstrated that the bacterial and human Lon variants not only recognize the shared substrates but also possess species-specific selectivity of substrates. For the convenience of description, the proteins digestible by only one Lon variant and by both eLon and hLon are hereafter referred to as unique and shared substrates, respectively.

### Lon cleavage sites in the shared substrates

Previous studies showed that both bacterial and human Lon variants predominantly cleave substrates at the hydrophobic amino acids although no specific amino acids or peptide sequences are identified as the recognition/cleavage sites^29,37,52^. We first tested whether different Lon variants have any unique preference in recognition and cleavage sites of their shared substrates by in vitro proteolysis and peptide identification using mass spectrometry (MS). For an unknown reason, the recombinant fLon was always tangled with bacterial genomic DNA, which prevented us from obtaining purified fLon with detectable enzymatic activity (not shown). In contrast, recombinant eLon and hLon were readily purified by affinity purification without the complication associated with fLon (see below).

We initially tested the cleavage of the three shared substrates: α-casein, RpsB, and SulA. α-casein is digestable by both eLon and hLon^53,54^. α-Casein was fully degraded within 2 h by both eLon and hLon (Fig. S2A), thus validating our in vitro degradation system. Under the same conditions, RpsB (Fig. S2B) and MBP-SulA (Fig. S2C) were also degraded by eLon and hLon. Further efforts were made to identify the peptides generated by in vitro proteolysis of these shared substrates using MS. The substrates were first treated with either eLon or hLon before being processed for peptide identification by liquid chromatography-tandem MS (LC-MS/MS). As listed in Table 1 and S5, treatment of α-casein, RpsB, and SulA with eLon and hLon yielded a large number of total and unique peptides. Relatively low levels of peptides were also identified in the control reaction without the protease, indicating spontaneous breakage of the peptide bonds.

**Table 1:** The numbers of Lon substrate-derived peptides identified in MS

Lon substrate-derived peptides^a^				
	α-Casein	RpsB	SulA	HUβ
eLon-treated				
Experiment 1	243	414	251	228
Experiment 2	217	295	195	245
Common^b^	111	245	159	186
hLon-treated				
Experiment 1	275	301	192	16
Experiment 2	206	204	172	19
Common^b^	138	135	115	10
Control				
Experiment 1	9	7	7	9
Experiment 2	10	6	13	20
Common^b^	3	3	4	8

^a^The number of peptide sequences that are unique to each Lon substrate

^b^The number of common peptides identified in two experiments

**Fig. 6:**
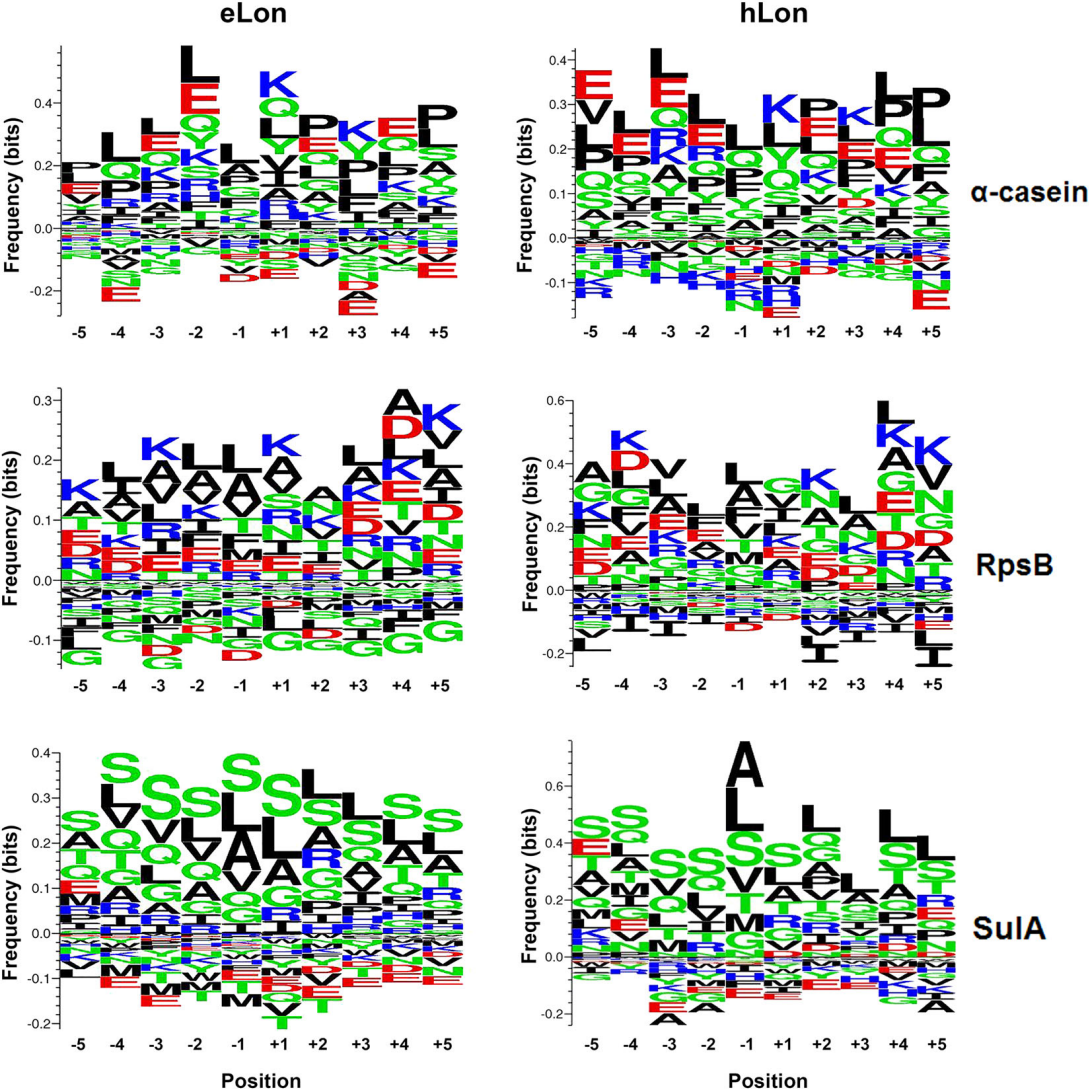
Web-based *Seq2logo* representation of amino-acid frequency at the Lon cleavage sites. The frequencies of the 10 amino acids (AAs) preceding (position −1) and following (position +1) all cleavage sites within α-casein, RpsB, and SulA were calculated as exemplified in Fig. S7B. The small and large sizes of the letters in each position represent relatively low and high frequencies of the corresponding amino acids, respectively. The AAs are presented as nonpolar and aliphatic group (black), polar and uncharged group (green), positively charged group (blue), or negatively charged group (red)

To define the cleavage sites of the Lon variants within each substrate, we analyzed 10 amino acids preceding (position −1) and following (position +1) all cleavage sites with a Python script. As represented in Fig. 6 and Fig. S3, the α-casein samples digested by both eLon and hLon showed a similar pattern of amino-acid preference at each of the 10 positions. Consistent with previous studies^29,37,52^, the position P(−1) residues at the cleavage sites of eLon and hLon were predominantly occupied by hydrophobic or nonpolar amino acids (e.g. leucine, alanine, valine, phenylalanine, and proline), among which leucine was the most abundant residue. In contrast, certain amino acids were rarely identified at this position of either protease-digested α-casein. As an example, only 3 of 25 glutamic acid residues in the protein was localized at the P(−1) position of eLon- or hLon-digested peptides (Fig. S3). Likewise, the 12 isoleucine residues within α-casein were either entirely absent (0, hLon) or marginally represented (1, eLon) at the same cleavage site. In a similar manner, the P(−1) position of the RpsB- and SulA-derived peptides were overwhelmingly represented by hydrophobic amino acids in the eLon- or hLon-treated samples. This is exemplified by leucine, alanine, and valine as the vast majority of the most abundant residues in all samples. Taken together, the MS results of the three common Lon substrates revealed a similar cleavage pattern between eLon and hLon, which was exemplified by predominant representation of leucine, alanine, and other hydrophobic amino acids at the P(−1) site within the substrates.

### Lon cleavage sites in HUβ

**Fig. 7:**
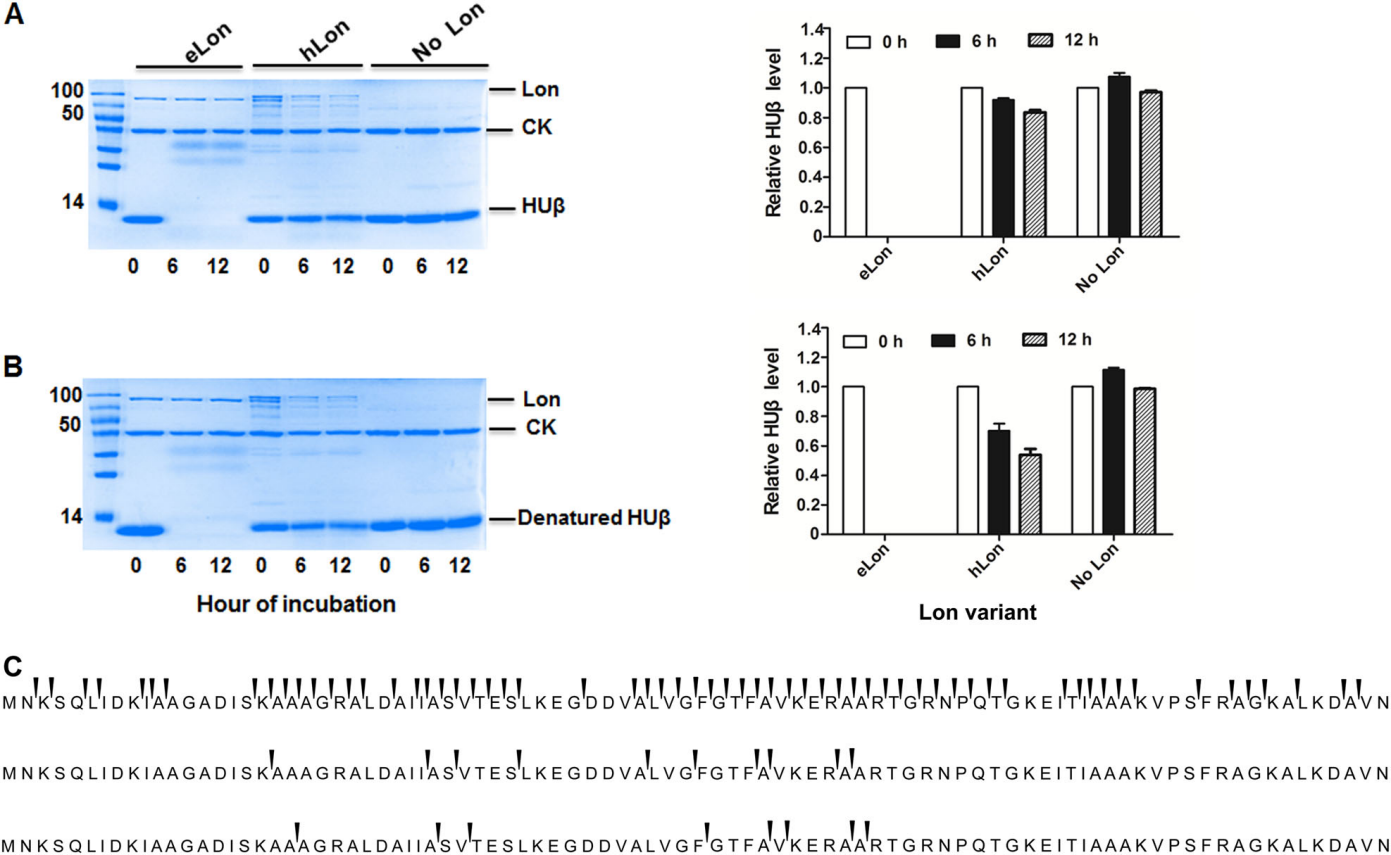
Differential degradation of HUβ by *E. coli* and human Lon variants. Recombinant HUβ (15 µg) was incubated at 37 °C with the Lon protease (10 µg) of *E. coli* (eLon) or human (hLon) before (**a**) or after (**b**) being denatured by heat. The proteins in the reactions were detected by SDS-PAGE and Coomassie Brilliant staining at 0, 6, and 12 h. Creatine kinase (CK) presented in the reaction mixture was used for ATP regeneration in this assay. HUβ was quantified by Image Lab and presented as relative value to the sample taken at 0 h (left panel of **a** and **b**). Sites of peptide bond break in HUβ in the presence of eLon (top panel) or hLon (middle), or in the absence of Lon protease (bottom) were identified by detection of peptides in the samples taken at 6 h by mass spectrometry (**c**). The arrows above the gaps of adjacent amino acids indicate the ends of individual peptides

We next tested the in vitro degradation of HUβ, a unique substrate for eLon, since it remained stable in vivo in the presence of hLon (Fig. 4). In agreement with our in vivo observation, HUβ became completely undetectable after 6 h incubation with eLon but not hLon under the in vitro conditions (Fig. 7a), thus confirming HUβ as a unique substrate for eLon. In the context of previous report that the structure or folded state of proteins is associated with Lon-mediated degradation^52^, these in vitro and in vivo proteolysis experiments raised a possibility that HUβ possesses a structure that is cleavable only by eLon. This possibility was tested by heat denaturing of purified HUβ before being treated with the Lon variants. As represented in Fig. 7b, heat-treated HUβ was readily degraded by eLon, but remained stable in the presence of hLon. To assess if HUβ possesses a relatively stable secondary structure that contributes to its resistance to hLon, we examined secondary structural change of HUβ after thermal denaturation at 96 °C by the circular dichroism (CD) spectrum (Fig. S8A). The CD spectrum of the untreated HUβ showed two negative peaks at 205 and 222 nm, indicative of α-helix structure(s), which agrees with what described in the previous study^29^. While these two peaks were obviously flattened in the heated HUβ, the extents of change were less dramatic as compared with the shifts in the control protein bovine serum albumin (BSA) samples (Fig. S8B). This result suggested that stable α-helix structure in HUβ contributes to its resistance to hLon.

We also tested another possibility that HUβ lacks a sequence tag or degron for hLon degradation. Previous studies showed that certain sequences or degrons facilitate the recognition and degradation of substrates by Lon^35^. We thus engineered a recombinant HUβ with the addition of sul20 peptide (a known Lon degron) at the carboxyl (C) end^33^ (Fig. S4A). Similar to the native HUβ, the tagged HUβ remained stable in the presence of hLon although it was degraded by eLon (Fig. S4B-D). We also attempted to enhance hLon degradation of HUβ by C-terminal tagging with various segments of RpsB (Fig. S4A), a substrate of hLon (Fig. 4). The sequences consisting of 20 (RpsB20), 30 (RpsB30), or 40 (RpsB40) amino acids surrounding the Val^163^ residue were chosen because it represented the most abundantly cleaved site in the hLon-treated RpsB (not shown). Surprisingly, the three RpsB-tagged HUβ variants were stable in the presence of eLon (Fig. S4C), suggesting that these sequence combinations disrupted the functional interaction of eLon with its natural substrate. Likewise, the RpsB-tagged HUβ proteins remained refractory to degradation by hLon (Fig. S4D).

Lastly, we determined the cleavage sites of HUβ by eLon. Recombinant HUβ was treated with eLon or hLon as described in Fig. 7a; the derived peptides identified by MS. In accordance to complete degradation of HUβ by eLon (Fig. 7), the two biological replicas each yielded more than 200 unique peptides with 186 peptides in common (Table 1); the protein showed a total of 62 cleavage sites (Fig. 7c, top panel). In sharp contrast, only 20 unique peptides were identified within the hLon-treated HUβ samples with 10 cleavage sites in total (Fig. 7c, middle panel), which is similar to the negative (no protease) controls (Fig. 7c, bottom panel). However, further comparison among the three sets of samples revealed that all the 10 cleavage sites in the hLon-treated HUβ were also identified in the eLon-treated protein, but only 2 sites of them overlap with those of the negative control. This result indicates that hLon shares certain cleavage sites within HUβ with eLon although its degradation efficiency is much lower.

### Impact of the Lon domains on substrate specificity

**Fig. 8:**
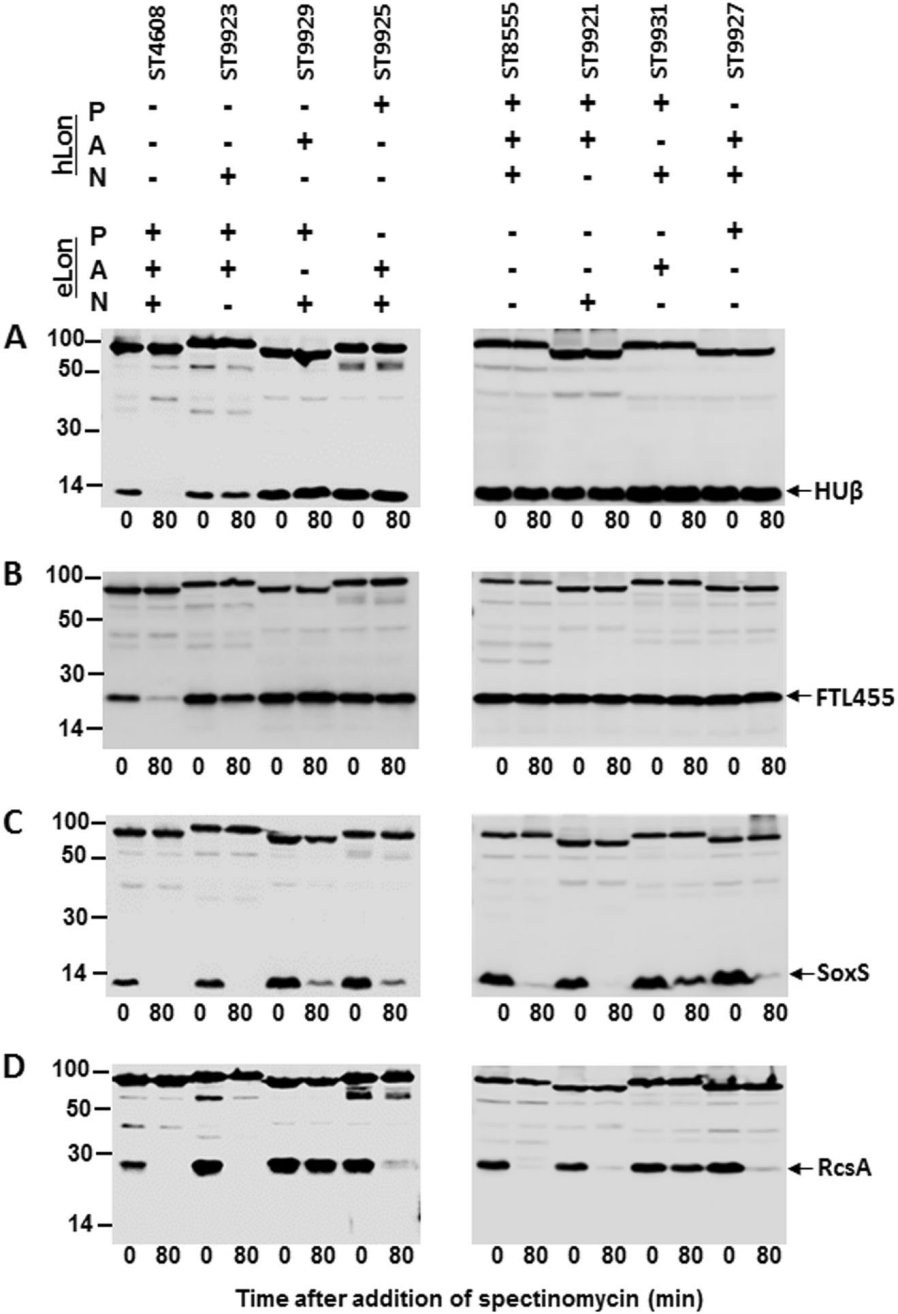
Degradation of representative Lon substrates by the Lon domain swap mutants or hybrid Lon variants. Stability of HUβ (**a**), FTL455 (**b**), SoxS (**c**), and RcsA (**d**) was detected in the presence of the domain swap mutants between eLon and hLon as illustrated in Fig. S5

Since the three domains of the LonA family proteases each fulfills a unique function in oligomerization and interactions with the substrate (N domain), ATP hydrolysis (A domain), and substrate degradation (P domain), we determined the individual contribution of the N, A, and P domains to the substrate specificity of the Lon variants. Rasulova et al.^55^ have demonstrated that the isolated proteolytic (P) domain of eLon exhibited proteolytic activity toward peptide substrate (26-amino-acid melittin). Moreover, fusing the N domain of eLon to the ClpX lacking its substrate recognition (N) domain successfully restored the degradation of the SsrA-tagged substrate Titin by ClpP (proteolytic subunit)^23^. These studies indicated that the proteolytic (P) and substrate recognition (N) domains of Lon do not functionally depend on the physical presence of the other domains, and are thus separable from the other domains. Thus, we constructed domain swap mutants of eLon and hLon, and each of the three domains in eLon and hLon was individually replaced by the counterpart in the other Lon variant to generate a series of the Lon hybrid proteins as outlined in Fig. S5. When tested for in vitro proteolysis of HUβ, a substrate of wild-type eLon but not hLon, the protein was effectively degraded by the wild-type eLon (Fig. 8a, construct ST4608). In contrast, the three Lon hybrids each with domain N (ST9923), A (ST9929), or P (ST9925) of hLon failed to degrade HUβ. Similar experiments did not detect any degradation of HUβ by either the wild-type hLon (ST8555) or its domain hybrids with eLon (Fig. 8a, right panel). This observation suggested that all of the three Lon domains are essential for the substrate specificity.

We further tested this notion with FTL455, another protein that was degraded by eLon but not by hLon (Fig. 3). Recombinant FTL455 was efficiently degraded by the wild-type eLon (Fig. 8b, left panel, second lane). Interestingly, the hybrid eLon with a hLon-N domain (construct ST9923) showed obvious but less effective degradation of FTL455 based on the observation that at the termination of the incubation (80 min), the amounts of the intact substrate left in the wild-type eLon and ST9923 reactions were 7 and 48% of the original amount, respectively (Fig. 8b, left panel, fourth lane; Fig. S6). However, replacing the A (ST9929) or P (ST9925) domain with the hLon counterpart led to complete loss of protease activity to FTL455 (Fig. 8b, left panel; Fig. S6). Similarly, none of the three hLon hybrids with the eLon domains digested FTL455 (Fig. 8b, right panel; Fig. S6). Together, hLon remained proteolytically inactive to both HUβ and FTL455 even after its three domains were individually replaced by the eLon counterparts.

To validate the importance of intrinsic match among the three domains of a single natural Lon variant in substrate degradation, we tested degradation of SoxS and RcsA, two shared substrates of eLon and hLon, by the Lon hybrids (Fig. 4). Consistent with the in vivo result (Fig. 4), purified recombinant SoxS was completely degraded by the wild-type eLon (Fig. 8c, ST4608). Replacing the N domain with that of hLon did not result in detectable effect on SoxS degradation (Fig. 8c, ST9923). However, similar constructs of domains A (ST9929, by 9%) and P (ST9925, by 11%) displayed substantial reduction in substrate degradation as compared with the wild-type eLon. In a similar manner, reciprocal domain replacements in hLon only led to minor changes in degradation of SoxS (Fig. 8c, right panel). The most significant reduction (by 14%) occurred in the A domain replacement construct (ST9931). A more dramatic impact of domain swap on the protease activity was observed with RcsA (Fig. 8d). The hLon-A domain replacement in eLon (ST9929) virtually abolished the protease activity of the wild-type eLon (by 96%). In a reciprocal fashion, the eLon-A domain swap in hLon (ST9931) also resulted in significant decrease (by 49%) in degradation of RcsA (Fig. 8d, right panel; Fig. S6). This result suggests that the A domain of the Lon proteases plays a vital role in substrate degradation. Taken together, these results demonstrated that all the three domains of the Lon proteases are necessary for substrate differentiation (or specificity) and degradation. These lines of evidence prompt us to conclude that substrate recognition and biochemical cleavage require intrinsic match of three domains in the Lon proteases.

## Discussion

The Lon protease performs many important functions in both prokaryotic and eukaryotic organisms, ranging from regulation of cellular morphology and virulence factors in bacteria to control of mitochondrial matrix protein quality and maintenance of mitochondrial DNA nucleoid integrity^1,20^. Dysfunction of hLon (LONP1) has been associated with numerous diseases such as aging^19^, cancer, and CODAS syndrome^20^. However, the molecular and biochemical mechanisms behind the substrate recognition and degradation of Lon remain largely obscure. The paucity of the knowledge on Lon biology is mirrored by the shortage of well-characterized substrate proteins for both prokaryotic and eukaryotic Lon variants^1,56^. On the basis of our previous discovery of five novel substrates of fLon^11^, this work has identified 10 additional new substrates of fLon, representing the largest number of the Lon substrates identified in a single study. Cross-species degradation of these and other known Lon substrates led to a surprising finding that the Lon variants of bacteria and humans can possess species specificity in substrate selection and degradation. Further analysis revealed that this organism-adapted substrate degradation depends on all of the three functional domains in the Lon proteases. The revelation of differential substrate degradation by the Lon variants provides a supporting evidence for engineering Lon and other proteases for medical and other applications in the future.

We identified a total of 10 new Lon substrates by a combination of proteomic, biochemical, and genetic approaches. To the best of our knowledge, this represents the largest number of Lon substrate proteins identified in a single study. This was accomplished by proteolysis screening of 21 candidates, which were previously obtained by quantitative proteomic comparison between *F. tularensis* LVS and its Lon-deficient mutant^11^. The high rate of success strongly suggests that our approach may be applied to substrate identification of Lon and other proteases in prokaryotic and eukaryotic organisms. The current study was based on the importance of the Lon protease in *F. tularensis* pathogenesis in our previous studies^11,57^. Although this study does not directly reveal the contribution of the newly identified Lon substrates to *F. tularensis* infection, this finding represents an important step toward the full understanding of molecular mechanisms governing the tularemia pathogenesis.

A major finding of this work is that the Lon variants in different organisms can adopt unique organism-adapted specificity in substrate selection. In our cross-species degradation profiling, a total of eight bacterial proteins (one eLon substrates and seven fLon substrates) were resistant to proteolysis by hLon. Indeed, the hLon showed relatively lower expression than bacterial Lon proteases in *E. coli*. This was likely due to the differences in codon usage between human and *E. coli*. However, we believe that the low expression of hLon did not significantly affect its substrate specificity because it readily degraded all of the three known natural substrates tested in this study (TFAM2, UNG1, and STAR) but not the negative control (GAPDH). In contrast, hLon degraded some (e.g. SoxS, SulA, and RcsA) but not the other (e.g. HUβ, FTL196, and FTL316) of the bacterial Lon substrates. Thus, we believe these differences reflect the natural variation of the Lon proteases in substrate recognition. While our data showed a sharp difference between bacterial and human Lon in substrate selection, a previous study has described a similar difference between the Lon variants of *E. coli* and *B. subtilis*. Ahn et al. reported that eLon does not degrade peroxide operon regulator PerR, a natural substrate of *B. subtilis* LonA^58^. To a lesser extent, eLon, fLon, and hLon also exhibited obvious differences in cleavage efficiency with some proteins that were degradable by all three proteases. For examples, SoxS was absolutely degradable by eLon and fLon, whereas 72% degraded by hLon. SulA was absolutely degraded by eLon but 57% degraded by fLon and 60% degraded by hLon, respectively (Fig. 4). Taken together, these observations strongly suggests a functional “micro co-evolution” or “organism adaptation” of the Lon variants with the biological context of their host organisms such that the structure and function of each Lon variant finely adapt to or match the substrates and biological need of the host organism.

The precise mechanism(s) of this “organism adaptation” phenomenon remains to be defined. In the context of the known modes of substrate selection by Lon, resistance of HUβ and fLon substrates to proteolysis by hLon likely to be caused by subtle sequence and structural variations in this Lon variant because both eLon and hLon variants degrade many shared substrates (e.g. α-casein, RpsB, and SulA) under the in vivo and in vitro conditions. In addition, the peptides generated by both eLon and hLon were demarcated at the P(−1) position by a similar set of amino acids, indicating that both the Lon variants share the basic principles in substrate recognition and cleavage. In other words, the resistance of the HUβ and *Francisella* proteins to proteolysis by hLon cannot be explained by the lack of cleavage sites within these proteins. Thus, it is reasonable to postulate that the inability of hLon to degrade bacterial Lon substrates occurs in the earlier step(s) in substrate selection (e.g. substrate recruitment and unfolding). Along this line, HUβ appears to adopt certain stable secondary structure(s), which prevents the protein from successful entry into the unfolding chamber of hLon. This notion is consistent with our observation that neither addition of the sul20 degron and other degradable sequences to HUβ nor heat denaturation made the protein cleavable by hLon.

The fine differentiation of HUβ by eLon and hLon is reminiscent of the dramatic difference in susceptibility of three bacterial histone-like proteins to proteolysis by eLon (i.e. HUα and HUβ of *E. coli*, and HU of *B. subtilis* or *Bs*-HU)^29^. HUα and *Bs*-HU share 68.9% and 52.2% amino-acid identity with HUβ, respectively. However, both HUα and *Bs*-HU are highly resistant to eLon, while HUβ is an endogenous substrate of the same protease. Ultimately, the organism-adapted substrate selectivity must lie in the sequence and structural variations among the Lon variants. Our domain swap experiments strongly suggested that this functional diversity is defined by sequence and structural differences in the three Lon domains because switching one or two domains between eLon and hLon did not revert their phenotypes in substrate selection. Furthermore, our results suggested that the A domain of the Lon proteases plays a vital role in substrate degradation. It is possible that the A domain locates at the central region of Lon, thereby readily affecting the structure and function of the entire protein. Complete understanding of the mechanisms governing the substrate selection of the Lon variants will await future structure-function comparison of the Lon variants, in terms of their molecular interactions with cleavable and close related non-cleavable proteins.

Studying how certain proteins are cleaved by certain Lon variants but not others will enable us to understand the protein-“killing” mechanisms of the Lon protease as a whole. Lon is the first ATP-dependent serine protease identified in *E. coli*^59^, but the molecular basis of its function is relatively less understood as compared with other members of the AAA+ protease family (e.g. ClpCP, ClpXP, and 26S proteosome). Arginine phophorylation promotes degradation of target proteins by ClpCP^60^. Post-translational addition of the SsrA tag to proteins marks them as substrates for degradation by ClpXP^61^. Likewise, polyubiquitination of target proteins marks them for degradation by the 26S proteosome in eukaryotes^62^. A recent study revealed that phosphorylation on *Xanthomonas citri* subsp. *Citri* Lon protects its target protein HrpG from degradation^63^. Redox switch of the two cysteine residues on the P domain of eLon also regulates Lon activity^64^. However, no unambiguous biochemical modification has been identified in the known Lon substrates. Genetic experiments have identified degrons in several substrates, including β-galactosidase^35^, UmuD^36^, SulA^37^, SoxS^32^, and SsrA^38^. A biochemical analysis revealed these degrons (e.g. sul20 and β20) can act as autonomous tags for native substrates degradation^35^. A comprehensive understanding of native substrate recognition by Lon has been elusive. In this context, comparative investigation of molecular interactions between the Lon variants and their substrates (e.g. bacterial histone-like proteins) can reveal valuable details on the principles governing substrate selection of Lon. While previous studies have provided structures of bacterial^21^ and human Lon^22^, no protease-substrate complex structures are available, which hinders the precise understanding of the substrate selection. The Lon substrates identified in this and our previous work^11^ have provided much more options for structural insight of the protease-substrate interactions.

Our identification of Lon variant- or species-specific substrates may promote future redesign of proteases or protease engineering. Because of their unique ability to cleave peptide bonds, proteases are widely used as research tools, detergent additives, and therapeutics. The clinically approved proteases are currently limited to those with naturally evolved specificities and catalytic properties, which are exemplified by clinical application of those associated with blood coagulation (e.g. factors IX and VIIa) and anti-coagulation (e.g. tissue plasminogen activator and urokinase-type plasminogen activator) blood clotting^65^. Recent advancement in engineering proteases has made it possible to generate new activities and specificities^49^. The major approaches of protease engineering consist of structure-guided design and random mutagenesis and screen^66–69^. The Lon substrates identified in this and our previous work^11^ may enable the structure biologists to obtain necessary information for structure-guided protease engineering of the Lon and other AAA+ proteases for new activities and specificities.

## Materials and methods

### Bacterial cultivation, plasmids, chemicals, and primers

*F. tularensis* LVS and its derivatives were cultured in Mueller-Hinton broth or on trypticase soy agar plates as described^11^. For protein induction, Chamberlain’s defined medium (CDM) was used instead^70^. When necessary, hygromycin (200 μg/ml) was added to the medium. *E. coli* strains were grown in Luria-Bertani (LB) broth or on LB agar plates at 37 °C in the presence or absence of ampicillin (100 μg/ml), chloramphenicol (34 μg/ml), or hygromycin (200 μg/ml). Unless stated otherwise, all bacterial media and chemicals were purchased from Sigma (Shanghai, China). All enzymes for DNA cloning were supplied by New England Biolabs (NEB) (Beijing, China). The bacterial strains and plasmids used in this study are listed in Table S1; the primers used in this study listed in Table S2.

### Expression constructs of recombinant proteins

Lon proteases were generated as C-terminally His-tagged recombinant proteins in pBAD18 as described in our previous study^11^. Briefly, the full eLon-expressing plasmid pST4608 was constructed by cloning the amplicon of primers Pr7265 and Pr7266 (from genomic DNA of *E. coli* MG1655) in the *Eco*RI/*Kpn*I site of pBAD18. The hLon construct pST8555 (lacking the first 114 amino acids of the predicted mitochondrial targeting sequence) was generated in the same manner by amplifying the coding region from a cDNA pool of HepG2 cells as described^71^. The enzymatic inactive Lon constructs with a mutation in the catalytic serine residue were generated essentially as described^72^. The fLon^S682A^ construct pST8438, containing an alanine in the position of serine 682, was constructed by primer-based PCR mutagenesis. The 3′ and 5′ flanking the serine codon was separately amplified from LVS genomic DNA; the fusion product of the two amplicons were generated by fusion PCR using primers Pr7259 and Pr7260, and cloned into the *Eco*RI/*Kpn*I site of pBAD18. In the same manner, primer sets of Pr7265/Pr11451 and Pr11450/Pr7266 (eLon^S679A^), and Pr11456/Pr11455 and Pr11454/Pr11302 (hLon^S855A^) were used to generate the eLon^S679A^ (pST8437) and hLon^S855A^ (pST8542) expression plasmids. Domain swap mutants of the Lon proteases were constructed in a similar fashion as described in Fig. S5. The resulting plasmids were verified by PCR amplification and DNA sequencing using primers Pr1423 and Pr1424.

The *Francisella* and *E. coli* proteins for in vivo degradation were prepared by cloning amplicons of the target coding sequences from the genomic DNA preparation of LVS and MG1655, respectively, and expressed as recombinant polypeptides with a C-terminal His tag in pACYCDuet-1 as described in our previous study^11^. The C-terminally peptide-tagged HUβ variants were generated as described previously^25^. The coding sequences of HUβ and each peptide were separately amplified from *E. coli* genomic DNA, linked by fusion PCR, and cloned into the *Nco*I/*Sal*I site of pACYCDuet-1. The specific primers and resulting constructs are described in Fig. S4A. The C-terminally His-tagged GAPDH was generated by cloning the coding region amplified from the cDNA pool of HepG2 cells in the *Nco*I/*Sal*I site of pACYCDuet-1. The codon-optimized coding sequences of STAR, TFAM2, and UNG1 were chemically synthesized (Synbio Tech, Suzhou, China) and cloned in the *Nco*I/*Sal*I site of pACYCDuet-1. The relevant primers and resulting plasmids are described in Table S3. The codon-optimized genes are available under the GenBank accessions MG824985 (TFAM2), MG824986 (UNG1), and MG824987 (STAR). The fLon substrates identified in *E. coli* were in *trans* expressed in LVS or its ∆*lon* mutant as His-tagged proteins in the *Mlu*I/*Xma*I site of pEDL17 as described^73^. The primers and resulting constructs are listed in Table S3.

### In vivo proteolysis

In vivo degradation of target proteins by the Lon proteases was evaluated in *E. coli* as described previously^11^. Briefly, the strains were grown to an OD_600_ of ~0.3 at 37 °C in LB broth, and 0.2% arabinose or sterile water (negative control) was added to induce one of the Lon proteases. After 2 h of expression, the putative Lon substrates or control proteins were induced with 1 mM IPTG for 0.5 h unless stated otherwise (the condition for production of *Francisella* recombinant protein are described in Table S3). Then the bacterial cells were harvested, washed once with LB medium, and resuspended in 1 culture volume of LB medium containing spectinomycin (100 μg/ml) to inhibit new protein synthesis as described^38^. A fraction of the cultures (1 ml) was removed at the indicated time points, pelleted, lysed, and immunoblotted. The target proteins were detected with anti-His_6_ monoclonal antibody (ZSGB-Bio, Beijing, China) as described previously^74^. Protein bands were visualized by the Clarity Western enhanced chemiluminescence reagent (Bio-Rad, Hercules, CA, USA) and immunoblot signals were quantified using Image Lab software (Bio-Rad) according to the supplier’s instructions.

Protein degradation in LVS was performed as described^38^. The resultant strains were cultivated to an OD_600_ of 0.6 in CDM broth before induction of target genes with 100 ng/ml ATc (Clontech, Mountain View, CA, USA) for 4 h with aeration. A fraction of the cultures (1 ml) was removed and pelleted by centrifugation for detection of target proteins by immunoblotting analysis using the anti-His_6_ antibody.

### In vitro proteolysis

The recombinant proteins used for the in vitro Lon degradation were purified from *E. coli* ER2566 derivatives containing the appropriate recombinant plasmids by affinity chromatography using the HisPur^TM^ Cobalt resins (Thermo, Waltham, MA, USA) as described^23^. Purified proteins were analyzed by SDS-polyacrylamide gel electrophoresis (SDS-PAGE); protein concentrations determined with the bicinchoninic acid assay kit (Solarbio, Beijing, China). The SulA carrying an N-terminal maltose-binding protein (MBP) was similarly purified with Amylose resins (NEB) as described^51^. Briefly, the coding region of *sulA* was amplified from *E. coli* MG1655 genomic DNA with primers Pr11809 and Pr11810, and cloned in the *Bam*HI/*Hin*dIII site of pMAL-p2X (NEB), resulting plasmid pST8967.

In vitro degradation was carried out as described^38^. For identification of cleavage sites in the Lon substrates, α-casein and purified RpsB, MBP-SulA, and HUβ were processed for degradation. Briefly, α-casein, RpsB, MBP-SulA, or HUβ (15 μg) was separately incubated with buffer, eLon, or hLon (10 μg) in 100 μl of buffer containing 25 mM Tris-HCl (pH 8.0), 100 mM KCl, 10 mM MgCl_2_, 1 mM dithiothreitol, and an ATP regeneration system [80 μg/ml creatine kinase (CK; Roche, Basel, Switzerland), 50 mM creatine phosphate (Roche), and 4 mM ATP]. The reaction mixtures were incubated at 37 °C and aliquots were taken at the indicated time points and analyzed by SDS-PAGE and Coomassie Brilliant staining as described previously ^74^.

Heat-denatured HUβ was prepared essentially as described^75^. Briefly, purified recombinant HUβ (~300 μg) was incubated at 96 °C for 15 min in 100 μl of Tris buffer (50 mM Tris and 300 mM NaCl, pH 8.0), cooled at room temperature for 30 min, and centrifuged at 12 000 rpm for 5 min. The supernatant was stored at −20 °C or immediately used for degradation.

### Mass spectrometry

Peptide identification from the Lon-treated protein samples was carried out essentially as described^76^. Briefly, each substrate protein (1 mg) was digested with eLon or hLon (100 µg) in a reaction volume of 1 ml for 6 h at 37 °C. The undigested substrate protein, Lon protease, and CK in the samples were removed using the Amicon Ultra Spin column (Millipore, Billerica, MA, USA); the resulting peptides purified using the C18 column (Waters, Milford, MA, USA) following the manufacturer’s instructions and analyzed by LC-MS/MS. As a negative control, the parallel reactions lacking the protease were also treated as described above. The MS/MS spectra from each LC-MS/MS run were searched against the respective protein sequences downloaded from UniProtKB (accession numbers: P02662 for α-casein; P0A7V0 for RpsB; P0AFZ5 for SulA; and P0ACF4 for HUβ) using in-house Sequest HT Algorithm in Proteome Discoverer software (version 1.4) with the following parameters: peptide MS tolerance of 20 ppm; MS/MS tolerance of 20 milli-mass units, carbamidomethylation of Cys as the fixed modification, deamidated on Asn and Gln, and oxidation on Met as the variable modification. Peptide spectral matches were validated using Percolator provided by Proteome Discoverer software based on *q* values at a 1% false-discovery rate.

### CD measurement

CD was performed as described^77^. BSA and purified HUβ (1 mg/ml) was incubated at 96 °C for 15 min in Tris buffer (10 mM Tris and 10 mM NaCl, pH 8.0), cooled at room temperature for 30 min, and centrifuged at 12 000 rpm for 5 min. The proteins in the supernatants were quantified and diluted to 0.2 mg/ml in sterile water. CD spectra were obtained with a Chirascan™-plus CD Spectrometer (Applied Photophysics, Leatherhead, U.K.). The spectra were recorded from 180 to 260 nm with a 0.1 cm light path in triplicate. Data were expressed as the means millidegrees.

### Bioinformatic analysis of substrate cleavage sites

Substrate cleavage site analysis was performed as described^52^. Substrate peptides identified from the MS data were matched to the corresponding protein sequences with custom-written Python 2.7 scripts. The Python 2.7 scripts were also used to count amino acids surrounding all cleavage sites. As depicted in Fig. S7A, when one peptide matched to its corresponding protein sequence, it revealed two cleavage sites. The first and last amino acids of the peptide denoted by the P(+1) and P(−1) site, respectively, and the surrounding amino acids denoted by P(−5) to P(−1) and P(+1) to P(+5) sites. The total number of amino-acid residues at the P(−5) to P(−1) and P(+1) to P(+5) positions represents the sum calculated through matching all peptides identified in MS.

The web-based *Seq2logo* was used for visualization of amino-acid profiles and frequencies^78^. The probability of amino acid in each position was calculated and submitted to *Seq2logo* in the frequency format (an example of the input is shown in Fig. S7B). In a *Seq2logo* output, the height of the bar is equal to the information content at each amino-acid position, the relative height of each individual amino acid is proportional to the bar. The information contents (bits) shown on *y*-axis is calculated using the relation *I* = ∑*p*_a_ · log_2_(*p*_a_/*q*_a_), where *p*_a_ and *q*_a_ refer to the observed frequency (included in the submitted data) and background frequency, respectively, of the amino acid a. When *p*_a_ < *q*_a_, the amino acid would be displayed on the negative *y*-axis.

## Electronic supplementary material

Supporting information

Table S5-1. Lon substrate α-casein-derived peptides identified in MS

Table S5-2. Lon substrate RpsB-derived peptides identified in MS

Table S5-3. Lon substrate SulA-derived peptides identified in MS

Table S5-4. Lon substrate HUβ-derived peptides identified in MS
