# Supplementary material for: Multiple domains of bacterial and human Lon proteases define substrate selectivity
Source: Emerg Microbes Infect. 2018 Aug 17;7:149. doi: 10.1038/s41426-018-0148-4 (PMC6098112; doi:10.1038/s41426-018-0148-4)
Supplement: Supplementary file 1 — Supporting information [file 41426_2018_148_MOESM1_ESM.pdf]

Multiple Domains of Bacterial and Human Lon Proteases Define Substrate Selectivity

Lihong He[1], Dongyang Luo[2], Fan Yang[3], Chunhao Li[4], Xuegong Zhang[2], Haiteng Deng[3], and Jing-Ren Zhang[1, 5*]

List of the supplementary figures and tables

Figure S1. Detection of protein stability in the presence of the proteolytically inactive Lon.

Figure S2. *In vitro* degradation of common substrates by the bacterial and human Lon variants.

Figure S3. Amino acid frequency at the cleavage sites within Lon common substrates.

Figure S4. Construction and *in vivo* degradation of the HUβ variants.

Figure S5. Schematic illustration for construction of the Lon proteases and their variants.

Figure S6. The amount of protein in the presence of the domain swap mutants between eLon and hLon.

Figure S7. Examples for amino acids surrounding Lon cleavage site and data input format in *Seq2logo.*

Figure S8. CD spectra of heat-treated and untreated HUβ and BSA.

Table S1. Bacterial strains and plasmids used in this study.

Table S2. Primers used in this study.

Table S3. The materials and conditions for production of recombinant target proteins.

Table S4. The biochemical characteristics of *Francisella* Lon substrates.

Table S5. Lon substrates α-casein-, RpsB-, SulA-, and HUβ-derived peptides identified in MS. (as individual files)

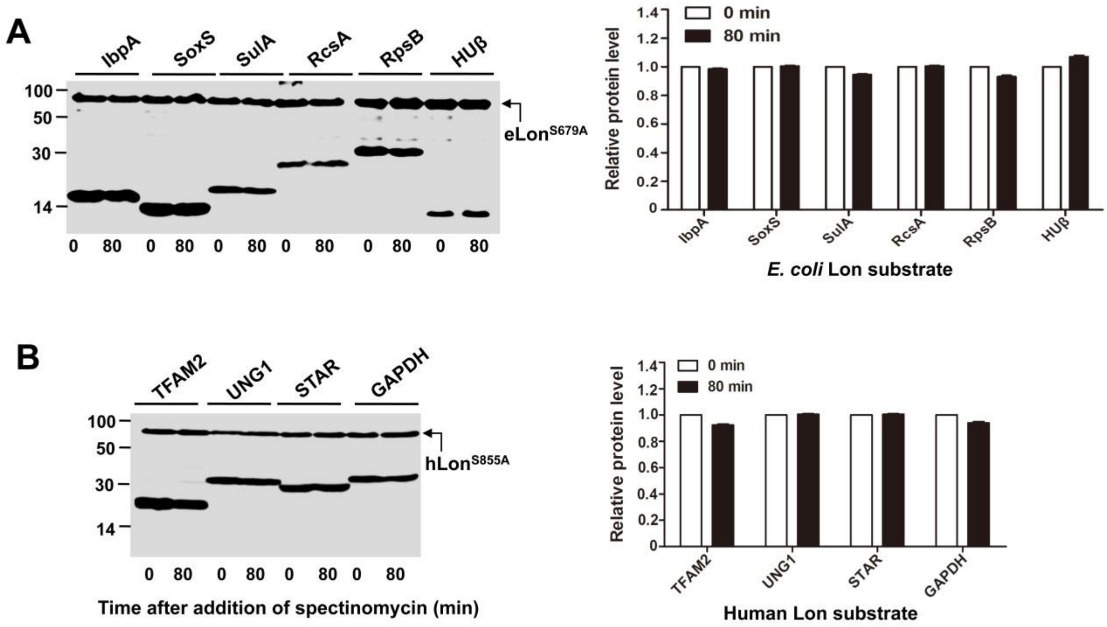

Figure S1. Detection of protein stability in the presence of the proteolytically inactive Lon. (A) Stability of the *E. coli* Lon substrates was detected in the presence of $eLon^{S679A}$. (B) Stability of the human Lon substrates was detected in the presence of $hLon^{S855A}$. Protein detection and quantification were carried out as in Fig. 1.

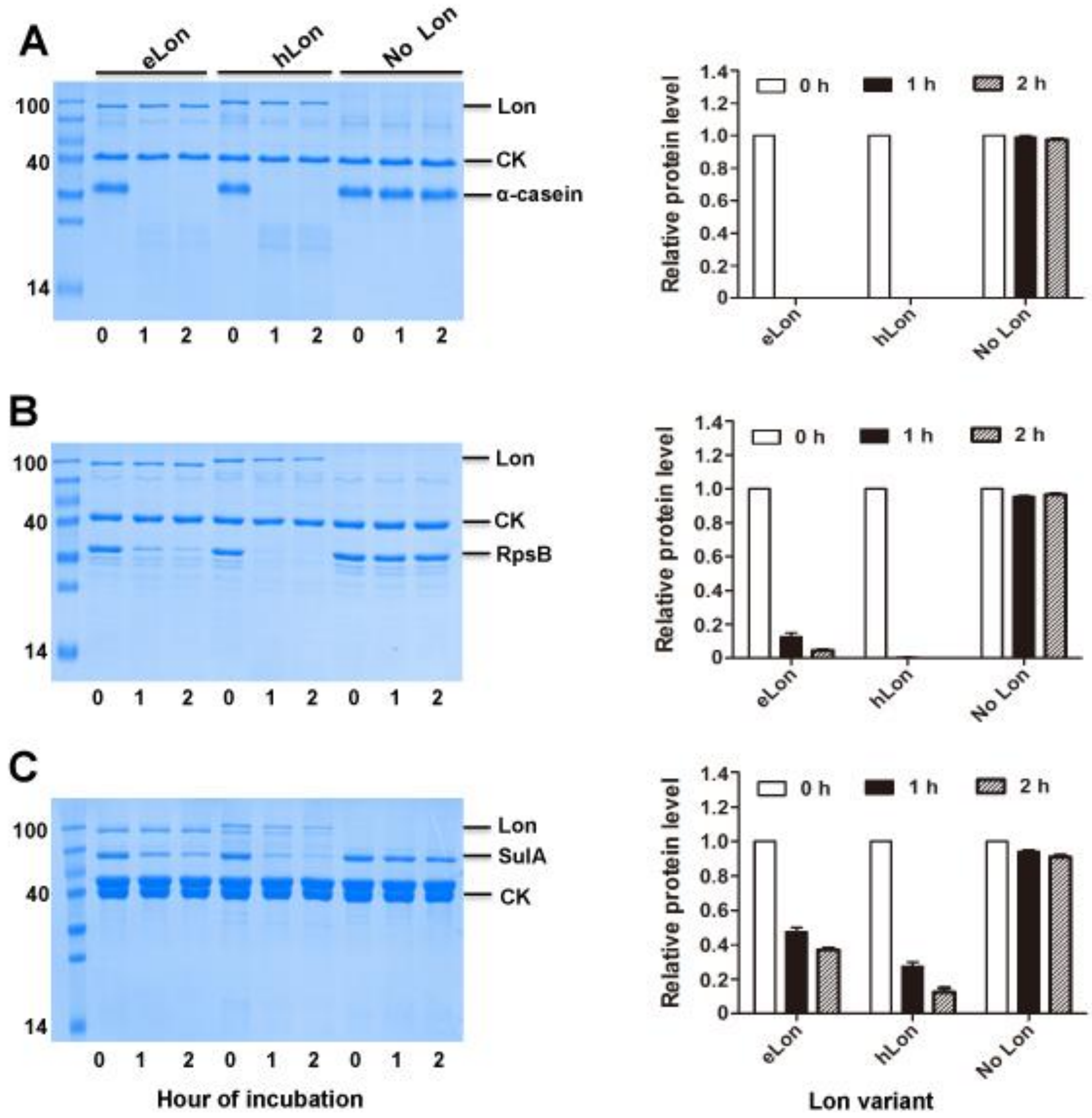

Figure S2. *In vitro* degradation of common substrates by the bacterial and human Lon variants. α-casein (A) and recombinant RpsB (B) and SulA (C) (15 μg) was incubated at 37 ℃ with the Lon protease (10 μg) of *E. coli* (eLon) or human (hLon). The proteins in the reactions were detected by SDS-PAGE and Coomassie Brilliant staining at 0, 1 and 2 h as in Fig. 7. Protein was quantified by Image Lab and presented as relative value to the sample taken at 0 hour as in Fig. 7 (left panel of A, B and C).

α-casein

eLon

amino acids	L	A	F	P	G	Q	V	D	K	Y	E	N	T	R	H	M	S	I	W	C	total
percentage	55	58	75	35	67	38	39	71	27	40	12	38	50	50	40	33	13	8	50	0	
-5	9	5	1	9	2	3	8	2	3	5	9	4	2	3	3	1	4	5	1	1	80
-4	14	3	4	9	2	9	4	1	2	3	7	4	1	4	2	3	4	4	0	0	80
-3	9	5	3	6	4	8	2	0	7	4	9	4	2	5	3	2	3	3	1	0	80
-2	14	1	4	1	3	7	3	1	6	6	12	1	3	5	4	0	5	3	0	1	80
Cleavage site → -1	12	7	6	6	6	5	5	5	4	4	3	3	3	3	2	2	2	1	1	0	80
+1	8	5	3	0	2	8	6	3	10	6	4	2	1	4	4	2	4	6	1	0	79
+2	7	6	4	12	6	7	5	2	5	5	8	2	1	2	3	1	2	1	1	0	80
+3	7	4	6	8	2	3	3	4	9	8	6	4	2	2	1	2	4	5	0	0	80
+4	8	5	4	7	4	8	4	2	6	4	10	2	2	2	1	1	5	3	2	0	80
+5	9	6	4	11	2	5	4	3	5	5	7	2	2	1	3	0	6	4	0	0	79
total residues	22	12	8	17	9	14	13	7	15	10	25	8	6	6	5	6	16	12	2	1	

hLon

amino acids	L	Q	F	P	G	Y	N	R	A	K	E	H	S	V	T	M	D	W	I	C	total
percentage	50	57	75	35	56	50	63	83	42	27	12	60	19	23	50	33	29	50	0	0	
-5	9	7	1	8	2	3	3	4	4	3	10	2	6	9	3	1	1	1	3	0	80
-4	11	7	4	8	5	2	4	4	4	3	10	3	2	4	1	3	1	0	4	0	80
-3	10	7	5	3	4	5	4	6	6	6	10	4	2	3	1	1	0	0	3	0	80
-2	13	6	5	6	3	5	2	6	2	4	9	4	4	3	3	0	1	1	3	0	80
-1	11	8	6	6	5	5	5	5	5	4	3	3	3	3	3	2	2	1	0	0	80
+1	9	8	4	2	2	8	3	4	3	10	4	4	5	2	1	3	2	0	5	0	79
+2	8	7	5	9	4	5	3	1	4	6	9	3	2	5	1	2	4	0	2	0	80
+3	8	4	6	7	4	5	3	3	4	9	8	2	3	3	2	1	5	0	3	0	80
+4	11	8	4	10	3	4	3	2	4	5	8	1	1	6	1	2	2	1	4	0	80
+5	10	6	6	11	4	4	4	2	5	2	7	3	4	3	1	1	2	0	4	0	79
total residues	22	14	8	17	9	10	8	6	12	15	25	5	16	13	6	6	7	2	12	1	

RpsB

eLon

amino acids	L	A	F	V	T	G	M	D	N	I	E	S	R	K	P	Q	H	Y	W	C	total
percentage	86	68	83	59	69	41	88	42	58	50	44	55	39	26	57	43	33	100	67	0	
-5	10	11	7	4	10	11	3	10	8	7	10	5	8	15	4	2	4	2	2	1	134
-4	14	11	5	10	9	9	4	10	6	11	9	4	5	10	5	5	4	2	2	0	135
-3	11	13	5	12	5	8	6	7	7	9	10	5	9	13	3	5	4	1	2	0	135
-2	17	13	10	10	6	6	5	6	7	10	9	6	7	9	5	4	2	2	1	0	135
Cleavage site → -1	18	17	10	10	9	7	7	7	7	7	7	6	5	5	4	3	2	2	2	0	135
+1	10	14	6	10	6	12	3	4	10	9	9	9	9	13	1	3	2	2	1	1	134
+2	8	12	2	10	9	10	5	8	11	9	8	7	6	11	5	5	3	2	2	1	134
+3	13	12	4	7	7	10	4	10	8	8	10	4	8	10	4	7	4	1	2	0	133
+4	11	13	6	8	9	10	2	12	8	5	10	3	8	11	7	3	3	1	2	0	132
+5	11	11	6	10	8	12	4	9	8	9	8	3	6	15	3	2	3	2	1	0	131
total residues	21	25	12	17	13	17	8	17	12	14	16	11	13	19	7	7	6	2	3	1	

hLon

amino acids	L	A	F	V	T	G	M	D	N	I	S	R	P	H	E	Y	Q	K	C	W	total
percentage	57	48	75	47	54	35	75	29	42	29	36	31	43	33	6	50	14	5	100	0	
-5	6	10	7	5	5	9	3	6	6	3	4	3	3	3	6	0	0	8	1	3	91
-4	8	6	7	6	5	8	2	9	5	8	1	2	3	4	6	1	2	9	0	0	92
-3	9	9	2	10	4	6	4	1	4	8	3	6	4	4	8	1	2	7	0	0	92
-2	10	7	10	6	3	6	2	4	4	8	5	6	1	2	9	1	3	4	0	1	92
-1	12	12	9	8	7	6	6	5	5	4	4	4	3	2	1	1	1	1	1	0	92
+1	8	7	2	9	2	10	2	5	5	9	4	6	1	2	7	2	2	7	0	1	91
+2	5	8	1	5	6	6	3	6	8	8	2	3	5	3	6	2	2	10	0	2	91
+3	11	10	4	3	5	6	4	6	7	7	1	5	2	3	5	1	2	7	0	2	91
+4	9	9	3	4	7	8	1	7	6	4	2	6	3	2	8	1	0	9	0	1	90
+5	6	6	1	10	5	6	3	6	9	8	1	5	2	3	4	2	0	11	0	1	89
total residues	21	25	12	17	13	17	8	17	12	14	11	13	7	6	16	2	7	19	1	3	

SulA

eLon

amino acids	A	L	S	V	G	Q	T	M	I	E	Y	F	H	W	K	N	D	R	P	C	total
percentage	100	67	67	64	67	46	46	72	67	40	40	100	33	50	25	20	25	11	17	100	
-5	9	5	11	5	2	8	8	6	4	7	2	1	2	2	4	4	1	5	5	0	91
-4	6	9	12	8	6	8	7	5	5	5	2	1	3	2	2	3	1	5	2	0	92
-3	5	7	18	9	7	8	5	5	4	5	3	2	1	1	3	3	1	4	2	0	93
-2	8	10	13	8	6	8	5	4	5	2	4	1	2	4	3	3	1	4	2	0	93
Cleavage site → -1	14	14	14	7	6	6	5	5	4	4	2	2	2	2	1	1	1	1	1	1	93
+1	9	15	15	5	7	4	5	3	5	3	3	2	2	2	1	2	3	5	1	0	92
+2	8	11	10	5	7	5	6	2	4	5	3	2	4	2	2	2	3	7	5	0	93
+3	7	13	12	6	2	7	5	1	6	5	3	1	4	3	3	2	3	3	5	1	92
+4	9	12	12	4	4	6	8	2	4	5	1	0	4	1	3	4	4	3	4	1	91
+5	9	11	12	5	2	5	7	3	3	5	1	1	3	1	3	5	3	6	5	1	91
total residues	14	21	21	11	9	13	11	7	6	10	5	2	6	4	4	5	4	9	6	1	

hLon

amino acids	A	L	S	V	T	M	G	E	N	I	H	F	D	Q	Y	W	K	P	R	C	total
percentage	93	57	48	64	55	72	56	30	40	33	33	100	25	8	20	0	0	0	0	0	
-5	6	4	10	5	6	4	3	7	3	3	1	1	1	5	0	2	4	2	3	0	70
-4	6	6	10	4	5	5	5	5	4	5	1	1	1	6	2	1	0	2	2	0	71
-3	5	6	12	7	5	5	4	4	2	1	2	2	0	7	3	1	3	1	2	0	72
-2	4	7	12	6	4	3	4	3	2	5	2	1	1	7	3	2	1	1	4	0	72
-1	13	12	10	7	6	5	5	3	2	2	2	2	1	1	1	0	0	0	0	0	72
+1	7	8	11	4	6	3	6	3	0	3	1	1	3	3	2	2	1	1	6	0	71
+2	6	11	4	4	4	2	6	4	2	4	0	1	3	6	3	1	2	5	4	0	72
+3	7	12	5	4	3	1	2	4	2	5	4	1	2	6	3	2	2	3	3	1	72
+4	6	12	9	3	6	2	3	2	2	4	3	0	3	4	1	1	3	4	3	0	71
+5	5	10	8	3	6	2	2	5	4	3	2	0	2	4	1	1	3	4	5	1	71
total residues	14	21	21	11	11	7	9	10	5	6	6	2	4	13	5	4	4	6	9	1	

Figure S3. Amino acid frequency at the cleavage sites within Lon common substrates. A Python script was used to calculate the amino acids surrounding the cleavage site. The total number of amino acid residues at the P (-5) to P (-1) and P (+1) to P (+5) positions within α-casein, RpsB, and SulA are shown in tabular form. Percentage refers to ratio of total number of amino acid residues at P (-1) position to that in α-casein, RpsB, and SulA protein sequence, respectively (shown as total residues). Total refers to the total number of amino acid residues in each position.

A

Tag fused to HUβ	Sequence	Primer set		Plasmid
		HUβ	Tag peptide	
sul20 (SulA$^{150-169}$)	ASSHATRQLSGLKIHSNLYH	Pr11813/Pr13599	Pr13600/Pr13601	pST11415
RpsB20 (RpsB$^{153-172}$)	DMGGLPDALF**V**IDADHEHIA	Pr11813/Pr13615	Pr13616/Pr13617	pST11416
RpsB30 (RpsB$^{148-177}$)	LGGIKDMGGLPDALF**V**IDADHEHIAIKEAN	Pr11813/Pr13731	Pr13732/Pr13620	pST11486
RpsB40 (RpsB$^{143-182}$)	KLENSLGGIKDMGGLPDALF**V**IDADHEHIAIKEANNLGIP	Pr11813/Pr13733	Pr13734/Pr13623	pST11488

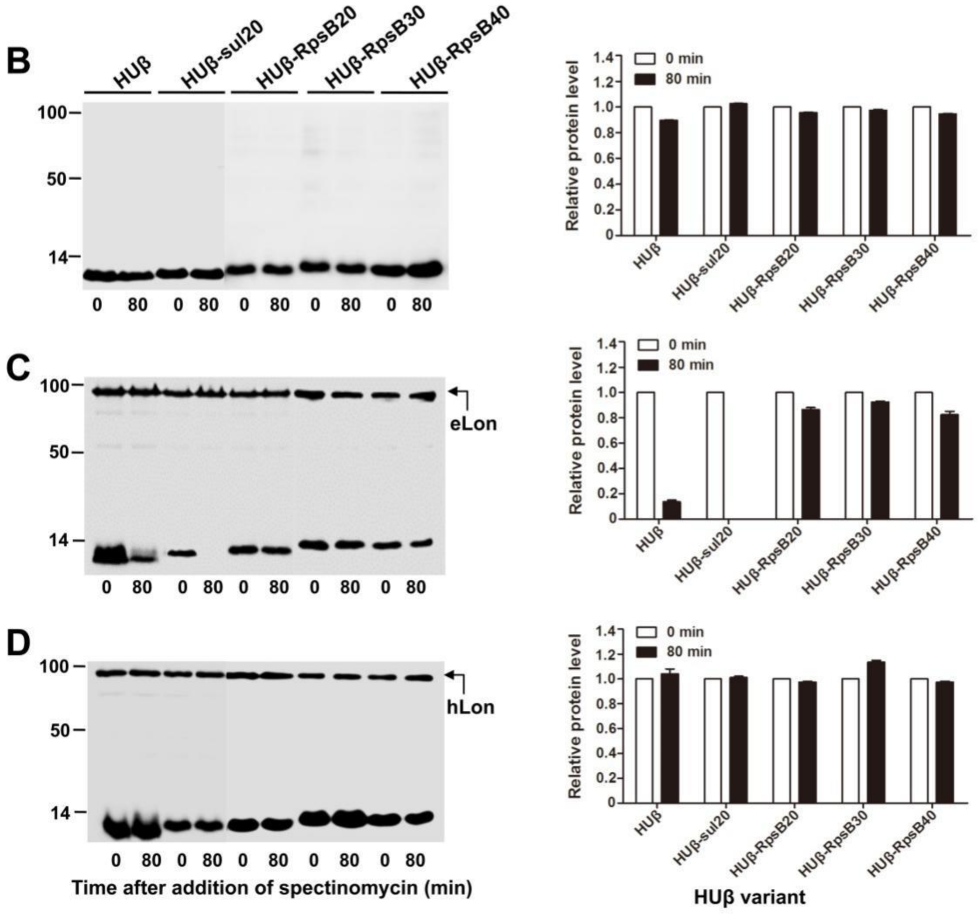

Figure S4. Construction and *in vivo* degradation of the HUβ variants. The tag sequences, primers and resulting plasmids were listed in (A). The bold-face letter shows amino acid Val^{163} which was the most abundantly cleaved site in the hLon-treated RpsB. Stability of the HUβ variants was detected in the absence (B) or presence of eLon (C) or hLon (D) as in Fig 3.

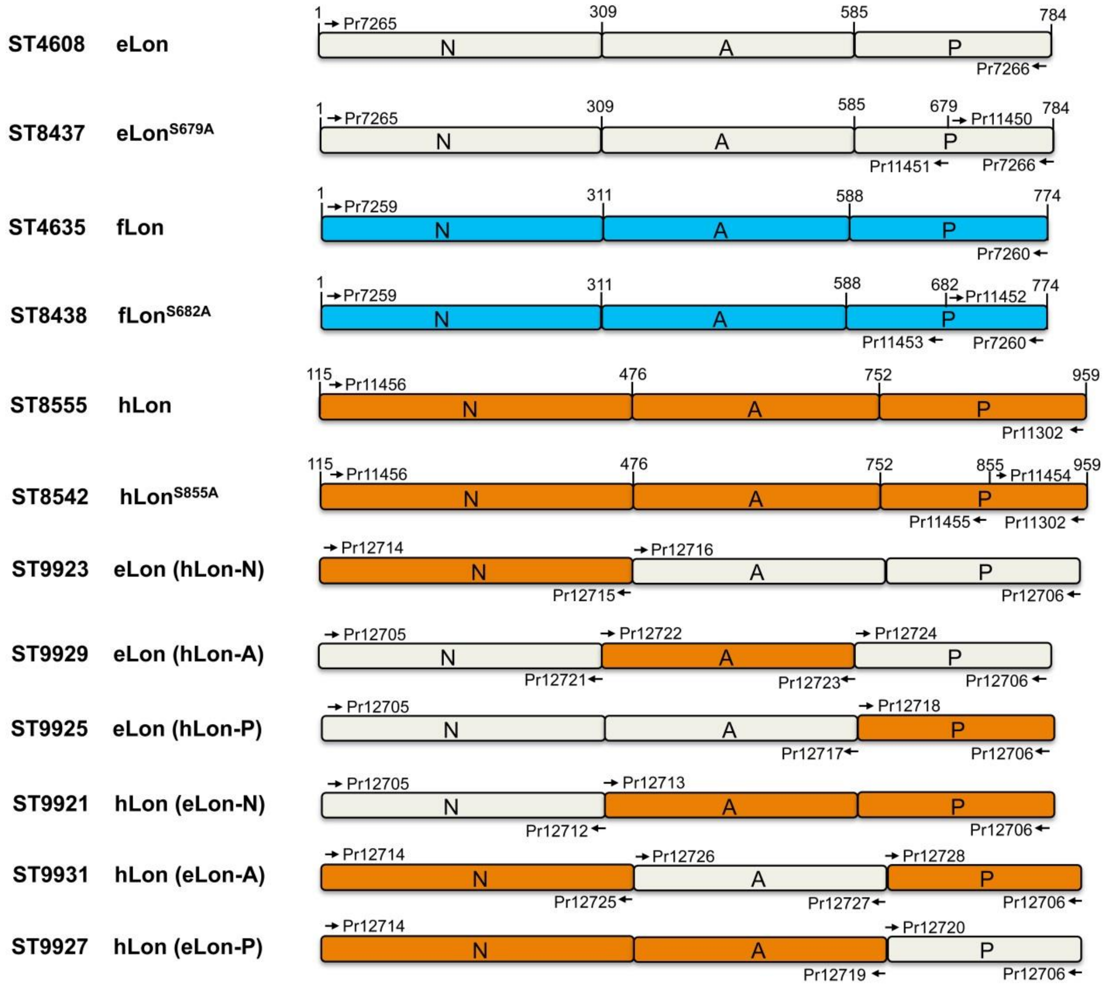

Figure S5. Schematic illustration for construction of the Lon proteases and their variants. Bacterial and human Lon consist of three domains: an N-terminal domain (N domain), a central ATPase domain (A domain) and a C-terminal proteolytic domain (P domain). These domains in eLon, fLon, or hLon are drawn in gray, blue, or orange, respectively. The range of each domain is indicated above the diagram. The hLon was constructed in truncated form lacking amino acids 1-114 of the predicted mitochondrial targeting sequence (MTS). The primer set used for construction of Lon proteases and their variants and the resulting plasmids are listed. The Lon domain swap mutants are designated following the principle, for example, eLon (hLon-N) represent that the N domain of eLon was replaced by the equivalent domain of hLon.

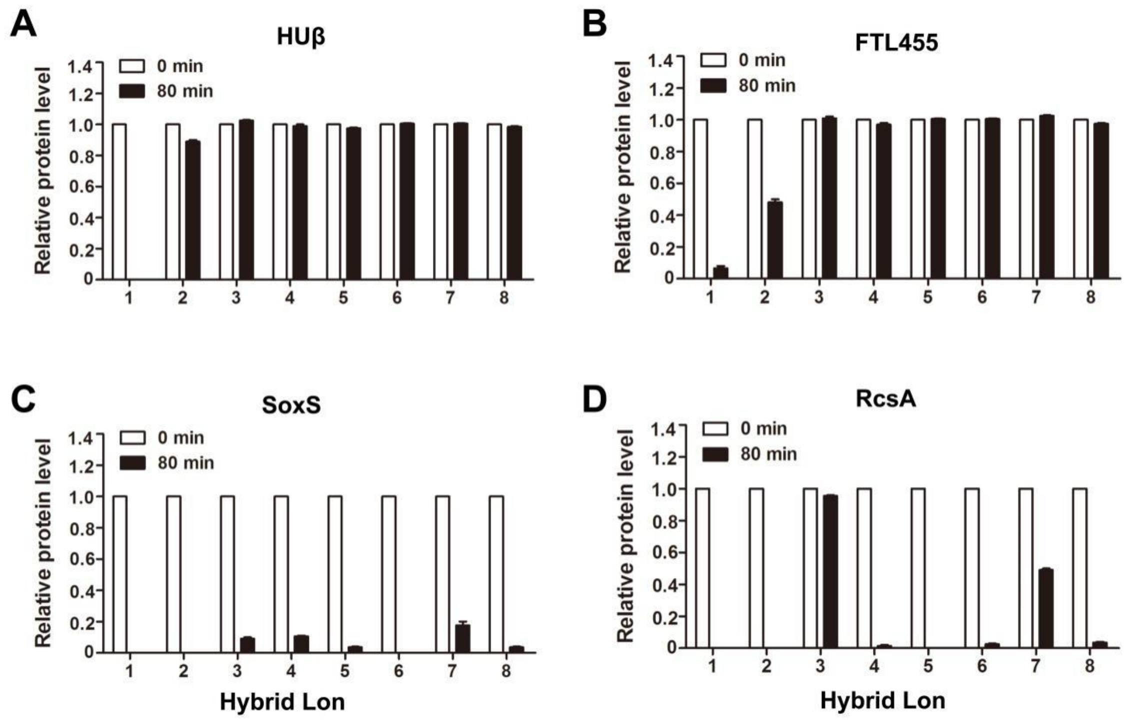

Figure S6. The amount of protein in the presence of the domain swap mutants between eLon and hLon. Bands from the Fig. 8 were quantified. The amount of HUβ (A), FTL455 (B), SoxS (C), and RcsA (D) were detected as in Fig. 1.

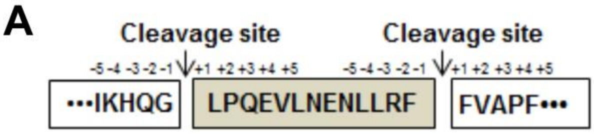

B

	L	A	F	P	G	Q	V	D	K	Y	E	N	T	R	H	M	S	I	W	C
-5	0.11	0.06	0.01	0.11	0.03	0.04	0.10	0.03	0.04	0.06	0.11	0.05	0.03	0.04	0.04	0.01	0.05	0.06	0.01	0.01
-4	0.18	0.04	0.05	0.11	0.03	0.11	0.05	0.01	0.03	0.04	0.09	0.05	0.01	0.05	0.03	0.04	0.05	0.05	0.00	0.00
-3	0.11	0.06	0.04	0.08	0.05	0.10	0.03	0.00	0.09	0.05	0.11	0.05	0.03	0.06	0.04	0.03	0.04	0.04	0.01	0.00
-2	0.18	0.01	0.05	0.01	0.04	0.09	0.04	0.01	0.08	0.08	0.15	0.01	0.04	0.06	0.05	0.00	0.06	0.04	0.00	0.01
-1	0.15	0.09	0.08	0.08	0.08	0.06	0.06	0.06	0.05	0.05	0.04	0.04	0.04	0.04	0.03	0.03	0.03	0.01	0.01	0.00
+1	0.10	0.06	0.04	0.00	0.03	0.10	0.08	0.04	0.13	0.08	0.05	0.03	0.01	0.05	0.05	0.03	0.05	0.08	0.01	0.00
+2	0.09	0.08	0.05	0.15	0.08	0.09	0.06	0.03	0.06	0.06	0.10	0.03	0.01	0.03	0.04	0.01	0.03	0.01	0.01	0.00
+3	0.09	0.05	0.08	0.10	0.03	0.04	0.04	0.05	0.11	0.10	0.08	0.05	0.03	0.03	0.01	0.03	0.05	0.06	0.00	0.00
+4	0.10	0.06	0.05	0.09	0.05	0.10	0.05	0.03	0.08	0.05	0.13	0.03	0.03	0.03	0.01	0.01	0.06	0.04	0.03	0.00
+5	0.11	0.08	0.05	0.14	0.03	0.06	0.05	0.04	0.06	0.06	0.09	0.03	0.03	0.01	0.04	0.00	0.08	0.05	0.00	0.00

Figure S7. Examples for amino acids surrounding Lon cleavage site and data input format in *Seq2logo.*One peptide from α-casein was listed and the amino acids surrounding cleavage site were labeled in (A). The frequency format of eLon-treated α-casein was presented in (B). It is characterized by having a header line containing all the amino acids, and a header column containing the given positions. The numbers in 20 columns represent the amino acid frequency in each position.

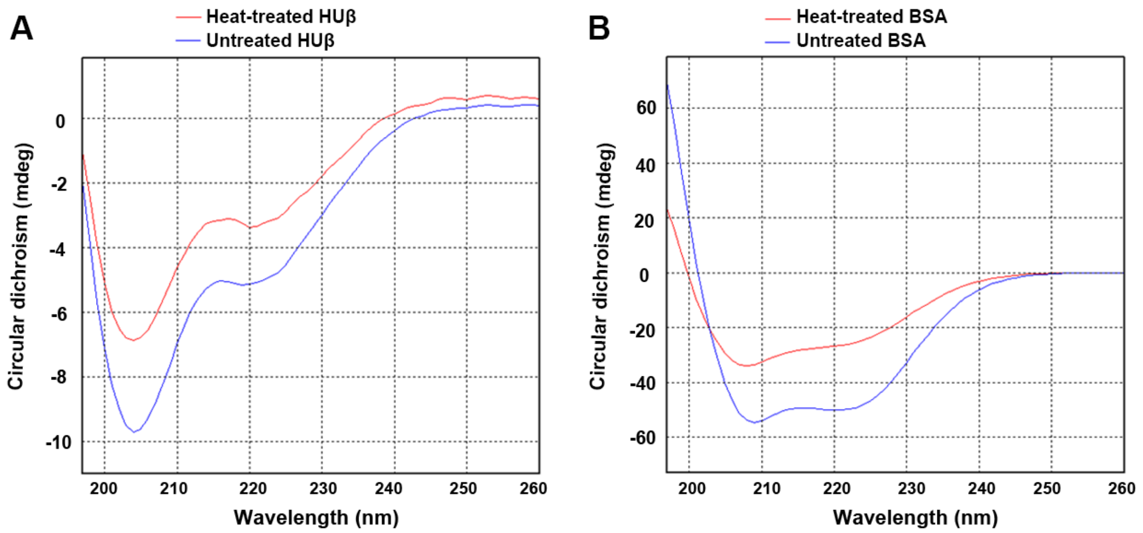

Figure S8. CD spectra of heat-treated and untreated HUβ and BSA. Spectra of heat-treated and untreated BSA and purified HUβ (0.2 mg/ml) in Tris buffer (2 mM Tris, 2 mM NaCl, pH 8.0) were recorded in a Chirascan™-plus CD Spectrometer. The red and blue lines represent the spectrum of heat-treated and untreated HUβ (A) and BSA (B), respectively.

Table S1. Bacterial strains and plasmids used in this study

Strain or plasmid	Description	Antibiotic resistance[1]	Reference or source
F. tularensis **subsp. *holarctica* strains**			
LVS	Live vaccine strain		[1]
ST1120	LVS derivative, Δ*lon*		This study
E. coli **strains**			
DH5α	F^- *endA1 glnV44 thi-1 recA1 relA1 gyrA96 deoR nupG* Φ80d*lacZ*ΔM15 Δ(*lacZYA-argF*)U169, *hsdR17*(r_K^- m_K^+), λ–		Biomed (Beijing, China)
ER2566	F- *λ- fhuA2 [lon] ompT lacZ::T7 gene 1 gal sulA11 Δ(mcrC-mrr)114::IS10R(mcr-73::miniTn10-TetS)2 R(zgb-210::Tn10)(TetS) endA1 [dcm]*		NEB
MG1655	*K-12 F– λ– ilvG– rfb-50 rph-1*		Biomed
**Plasmids**			
pACYCDuet-1	*E. coli* protein expression vector	Cm^R	Novagen
pBAD18	*E. coli* protein expression vector	Amp^R	[2]
pEDL17	*Francisella-E. coli* shuttle plasmid	Hyg^R	[3]
pST4608	pBAD18::eLon	Amp^R	This study
pST4609	pACYCDuet-1::FTL663	Cm^R	[4]
pST4611	pACYCDuet-1::FTL1957	Cm^R	[4]
pST4635	pBAD18::fLon	Amp^R	[4]
pST5005	pACYCDuet-1::FTL127	Cm^R	This study
pST5007	pACYCDuet-1::FTL578	Cm^R	[4]
pST5009	pACYCDuet-1::FTL1217	Cm^R	[4]
pST5010	pACYCDuet-1::FTL1218	Cm^R	This study
pST7524	pACYCDuet-1::FTL160	Cm^R	This study
pST7525	pACYCDuet-1::FTL196	Cm^R	This study
pST7526	pACYCDuet-1::FTL316	Cm^R	This study
pST7527	pACYCDuet-1::FTL455	Cm^R	This study
pST7528	pACYCDuet-1::FTL499	Cm^R	This study
pST7529	pACYCDuet-1::FTL528	Cm^R	This study
pST7530	pACYCDuet-1::FTL714	Cm^R	This study
pST7531	pACYCDuet-1::FTL964	Cm^R	This study
pST7532	pACYCDuet-1::FTL965	Cm^R	This study
pST7533	pACYCDuet-1::FTL975	Cm^R	This study
pST7534	pACYCDuet-1::FTL995	Cm^R	This study
pST7535	pACYCDuet-1::FTL1003	Cm^R	This study
pST7536	pACYCDuet-1::FTL1034	Cm^R	This study
pST7537	pACYCDuet-1::FTL1136	Cm^R	This study

pST7538	pACYCDuet-1::FTL1167	Cm^R	This study
pST7539	pACYCDuet-1::FTL1216	Cm^R	This study
pST7540	pACYCDuet-1::FTL1244	Cm^R	This study
pST7541	pACYCDuet-1::FTL1566	Cm^R	This study
pST7542	pACYCDuet-1::FTL1644	Cm^R	This study
pST7543	pACYCDuet-1::FTL1917	Cm^R	This study
pST7544	pACYCDuet-1::FTL1923	Cm^R	This study
pST7545	pACYCDuet-1::FTL1935	Cm^R	This study
pST7975	pEDL17::FTL196	Hyg^R	This study
pST7976	pEDL17::FTL316	Hyg^R	This study
pST7977	pEDL17::FTL455	Hyg^R	This study
pST7978	pEDL17::FTL964	Hyg^R	This study
pST7979	pEDL17::FTL1003	Hyg^R	This study
pST7980	pEDL17::FTL1034	Hyg^R	This study
pST7981	pEDL17::FTL1167	Hyg^R	This study
pST7982	pEDL17::FTL1216	Hyg^R	This study
pST7983	pEDL17::FTL1935	Hyg^R	This study
pST7984	pEDL17::FTL1218	Hyg^R	This study
pST7985	pEDL17::FTL965	Hyg^R	This study
pST7986	pEDL17::FTL995	Hyg^R	This study
pST7987	pEDL17::FTL1566	Hyg^R	This study
pST7988	pACYCDuet-1::IbpA	Cm^R	This study
pST7989	pACYCDuet-1::SoxS	Cm^R	This study
pST7990	pACYCDuet-1::SulA	Cm^R	This study
pST7991	pACYCDuet-1::RcsA	Cm^R	This study
pST8437	pBAD18::$eLon^{S679A}$	Amp^R	This study
pST8438	pBAD18::$fLon^{S682A}$	Amp^R	This study
pST8542	pBAD18::$hLon^{S855A}$	Amp^R	This study
pST8555	pBAD18::hLon	Amp^R	This study
pST8721	pACYCDuet-1::GAPDH	Cm^R	This study
pST8967	pMAL-p2X::SulA	Amp^R	This study
pST8968	pACYCDuet-1::RpsB	Cm^R	This study
pST8969	pACYCDuet-1::HUβ	Cm^R	This study
pST9320	pACYCDuet-1::TFAM2	Cm^R	This study
pST9321	pACYCDuet-1::UNG1	Cm^R	This study
pST9322	pACYCDuet-1::STAR	Cm^R	This study
pST9921	pBAD18::hLon (eLon-N)	Amp^R	This study
pST9923	pBAD18::eLon (hLon-N)	Amp^R	This study
pST9925	pBAD18::eLon (hLon-P)	Amp^R	This study
pST9927	pBAD18::hLon (eLon-P)	Amp^R	This study
pST9929	pBAD18::eLon (hLon-A)	Amp^R	This study

pST9931	pBAD18::hLon (eLon-A)	Amp^R	This study
pST11415	pACYCDuet-1::HUβ-sul20	Cm^R	This study
pST11416	pACYCDuet-1::HUβ-RpsB20	Cm^R	This study
pST11486	pACYCDuet-1::HUβ-RpsB30	Cm^R	This study
pST11488	pACYCDuet-1::HUβ-RpsB40	Cm^R	This study

[1]Antibiotic resistance: Amp^R, ampicillin; Cm^R, chloramphenicol; Hyg^R, hygromycin.

Table S2. Primers used in this study

Primer	Sequence (5’-3’)
Pr1423	TACCTGACGCTTTTTATCGCAACT
Pr1424	GAGTTCGGCATGGGGTCAG
Pr7259	CGGAATTCAGGAGGATTACATATGTCAGAACCCCTAAATGTCGTT
Pr7260	GGGGTACCCTAATGATGATGATGGTGATGAAAAACTCTCTCTAAAACCTCTT
Pr7265	CGGAATTCAGGAGGATTACATATGAATCCTGAGCGTTCTGAACG
Pr7266	GGGGTACCCTAATGATGATGATGGTGATGTTTTGCAGTCACAACCTGCA
Pr7753	CATGCCATGGGCCATAATGATATTAGCGAGTATTT
Pr7754	GCGTCGACCTAATGATGATGATGGTGATGTATAATTTTCTTATTTGTATCTAA
Pr7757	CATGCCATGGGCAAGATATTATGTATTTTATATG
Pr7758	CGGAATTCCTAATGATGATGATGGTGATGACTATAAGTTAAATTGAGCTCTT
Pr10874	CATGCCATGGGCCTTTTAATAAATTGTGATTTAGGA
Pr10875	CGGAATTCCTAATGATGATGATGGTGATGAAAGCAATCCTCACCAGCAATTT
Pr10876	CATGCCATGGGCCAATACACTCTAAAACAAATATCC
Pr10877	CGGAATTCCTAATGATGATGATGGTGATGAAGATCTATGTTGAGGCTTTTAGC
Pr10878	CATGCCATGGGCATAAAAGTATTTGGAATAAATAAT
Pr10880	CATGCCATGGGCAATTTTAATAAATCTCTCTACTAT
Pr10881	CGGAATTCCTAATGATGATGATGGTGATGATCTAAATTATTACTACTTTGCC
Pr10882	CATGCCATGGGCATCATTCAAAATACTACAGCTATC
Pr10883	CGGAATTCCTAATGATGATGATGGTGATGATTTACTACCTCGACTACTTCACC
Pr10884	CATGCCATGGGCCTATTTGAAAAACAACAGTATCAA
Pr10886	CATGCCATGGGCAGCCAGCTATCTCTTAATAAAAAG
Pr10887	GCGTCGACCTAATGATGATGATGGTGATGTACTAAATAACGGGCTTTTATGG
Pr10888	CATGCCATGGGCACACAAATAATGACTCCAAAAAC
Pr10889	CGGAATTCCTAATGATGATGATGGTGATGTAGAATATATTGACTTAGATCTTT
Pr10890	CATGCCATGGGCGAAGCAATTAAAGGAACTACTATT
Pr10891	CGGAATTCCTAATGATGATGATGGTGATGTCCTTTTTTATTTTCTAAACTTTC
Pr10892	CGGGATCCGGCTTTAAAATTGCAGATATAGA
Pr10893	GCGTCGACCTAATGATGATGATGGTGATGAAATTTATATAATGATTTTTTATAC
Pr10894	CATGCCATGGGCCTTTTACCTGTACATAAAAATAC
Pr10895	CGGAATTCCTAATGATGATGATGGTGATGATCTAACTCAATATTTCTAACATC
Pr10896	CATGCCATGGGCATATATATACTATTTTTTAGAATG
Pr10897	CGGAATTCCTAATGATGATGATGGTGATGCCATTTTGCATCTTCTTCTAGTTT
Pr10898	CATGCCATGGGCTCACATAAGTCTGATTTAATTGC
Pr10900	CATGCCATGGGCCAAATTCATATAATTGATATTCAC
Pr10902	CATGCCATGGGCTTAAATATTATAAATGACTCCTTA
Pr10903	CGGAATTCCTAATGATGATGATGGTGATGAGATGTTTTTACATTTATTTGTCC
Pr10904	CATGCCATGGGCTATCAAAATCATAAAAGTGAAATT
Pr10905	CGGAATTCCTAATGATGATGATGGTGATGGCAATTACCTGTATTTCTATTAA

Pr10908	CATGCCATGGGCACAAACTTAGAAATATGTGTAGA
Pr10909	CGGAATTCCTAATGATGATGATGGTGATGATTGTTAAGCTTTGATTTTATTG
Pr10910	CATGCCATGGGCTCAAAAGATTTCATCTTAGCTATAG
Pr10911	GCGTCGACCTAATGATGATGATGGTGATGACTATAACACTCTGCCCAAGCCTT
Pr10912	CATGCCATGGGCAAACCTTTAAAAATACTTATCGAT
Pr10913	CGGAATTCCTAATGATGATGATGGTGATGTCGATTTTGTATCATCAATAACCA
Pr10914	CATGCCATGGGCCCTGCGCAATATCACATTGGAAC
Pr10915	CGGAATTCCTAATGATGATGATGGTGATGTGTTGTAGTTGTATACTCTGCGG
Pr10916	CATGCCATGGGCATTAAATGCTCTAACTTGGTGATT
Pr10917	CGGAATTCCTAATGATGATGATGGTGATGAACACAAATATCCTCCTGTAAACA
Pr10928	CATGCCATGGGCGTAATGACTTTTAATGCCAATGGT
Pr10929	GCGTCGACCTAATGATGATGATGGTGATGAACCTCATAATCATAATTAATAG
Pr10930	GCGTCGACCTAATGATGATGATGGTGATGACCCCATTTTTCTTCATACTCTTT
Pr10931	GCGTCGACCTAATGATGATGATGGTGATGTTTTTGGTACTCCTGTAAAATATT
Pr10932	GCGTCGACCTAATGATGATGATGGTGATGTATCCCCCTACTAATATCTATTTC
Pr10933	GCGTCGACCTAATGATGATGATGGTGATGTGAGTTAGCAAGTTCTGGGATATG
Pr11189	CGACGCGTTTGCAATACACTCTAAAACAAATATCC
Pr11190	CCCCCCGGGCTAATGATGATGATGGTGATGAAGATCTATGTTGAGGCTTTTAGC
Pr11191	CGACGCGTATGATAAAAGTATTTGGAATAAATAAT
Pr11192	CCCCCCGGGCTAATGATGATGATGGTGATGACCCCATTTTTCTTCATACTCTTT
Pr11193	CGACGCGTATGAATTTTAATAAATCTCTCTACTAT
Pr11194	CCCCCCGGGCTAATGATGATGATGGTGATGATCTAAATTATTACTACTTTGCC
Pr11195	CGACGCGTATGACACAAATAATGACTCCAAAAAC
Pr11196	CCCCCCGGGCTAATGATGATGATGGTGATGTAGAATATATTGACTTAGATCTTT
Pr11197	CGACGCGTATGATATATATACTATTTTTTAGAATG
Pr11198	CCCCCCGGGCTAATGATGATGATGGTGATGCCATTTTGCATCTTCTTCTAGTTT
Pr11199	AGTGGCGCGCCATGTCACATAAGTCTGATTTAATTGC
Pr11200	CCCCCCGGGCTAATGATGATGATGGTGATGTATCCCCCTACTAATATCTATTTC
Pr11201	CGACGCGTATGTTAAATATTATAAATGACTCCTTA
Pr11202	CCCCCCGGGCTAATGATGATGATGGTGATGAGATGTTTTTACATTTATTTGTCC
Pr11203	CGACGCGTATGTATCAAAATCATAAAAGTGAAATT
Pr11204	CCCCCCGGGCTAATGATGATGATGGTGATGGCAATTACCTGTATTTCTATTAA
Pr11205	AGTGGCGCGCCATGATTAAATGCTCTAACTTGGTGATT
Pr11206	CCCCCCGGGCTAATGATGATGATGGTGATGAACACAAATATCCTCCTGTAAACA
Pr11207	CGACGCGTATGCATAATGATATTAGCGAGTATTT
Pr11208	CCCCCCGGGCTAATGATGATGATGGTGATGTATAATTTTCTTATTTGTATCTAA
Pr11209	CGACGCGTATGGAAGCAATTAAAGGAACTACTATT
Pr11210	CCCCCCGGGCTAATGATGATGATGGTGATGTCCTTTTTTATTTTCTAAACTTTC
Pr11211	CGACGCGTATGCTTTTACCTGTACATAAAAATAC
Pr11212	CCCCCCGGGCTAATGATGATGATGGTGATGATCTAACTCAATATTTCTAACATC
Pr11213	CGACGCGTATGACAAACTTAGAAATATGTGTAGA

Pr11214	CCCCCCGGGCTAATGATGATGATGGTGATGATTGTTAAGCTTTGATTTTATTG
Pr11215	CATGCCATGGGCCGTAACTTTGATTTATCCCCGCT
Pr11216	GCGTCGACCTAATGATGATGATGGTGATGGTTGATTTCGATACGGCGCGGTT
Pr11217	CATGCCATGGGCTCCCATCAGAAAATTATTCAGGA
Pr11218	GCGTCGACCTAATGATGATGATGGTGATGCAGGCGGTGGCGATAATCGCTGG
Pr11219	CATGCCATGGGCTACACTTCAGGCTATGCACATCG
Pr11220	GCGTCGACCTAATGATGATGATGGTGATGATGATACAAATTAGAGTGAATTT
Pr11221	CATGCCATGGGCTCAACGATTATTATGGATTTATG
Pr11222	GCGTCGACCTAATGATGATGATGGTGATGGCGCATGTTGACAAAAATACCAT
Pr11302	GGGGTACCCTAATGATGATGATGGTGATGTTCCACGGCCAGCGCCTCTGC
Pr11450	CGACGCCGAAAGATGGTCCGGCTGCCGGTATTGCTATGTGCA
Pr11451	TGCACATAGCAATACCGGCAGCCGGACCATCTTTCGGCGTCG
Pr11452	CTACACCAAAAGATGGTCCAGCTGCTGGTATTGCGATGACAA
Pr11453	TTGTCATCGCAATACCAGCAGCTGGACCATCTTTTGGTGTAG
Pr11454	CCACCCCCAAGGACGGCCCAGCCGCAGGCTGCACCATCGTCA
Pr11455	TGACGATGGTGCAGCCTGCGGCTGGGCCGTCCTTGGGGGTGG
Pr11456	CGGAATTCAGGAGGATTACATATGACGATCCCCGATGTGTTTCCGCAC
Pr11626	CGGAATTCATGGGGAAGGTGAAGGTCGGAG
Pr11627	GCGTCGACCTAATGATGATGATGGTGATGCTCCTTGGAGGCCATGTGGG
Pr11809	CGGGATCCATGTACACTTCAGGCTATGCACAT
Pr11810	CCAAGCTTTTAATGATACAAATTAGAGTGAATT
Pr11811	CATGCCATGGGCGCAACTGTTTCCATGCGCGACATG
Pr11812	GCGTCGACCTAATGATGATGATGGTGATGCTCAGCTTCTACGAAGCTTTCT
Pr11813	CATGCCATGGGCGTGAATAAATCTCAATTGATCGAC
Pr11814	GCGTCGACCTAATGATGATGATGGTGATGGTTTACCGCGTCTTTCAGTGCT
Pr12088	CATGCCATGGGCGCTTTTCTGCGTAGTATGTGGG
Pr12089	GCGTCGACCTAATGATGATGATGGTGATGACACTCTTCTGCGCCGTATT
Pr12090	CATGCCATGGGCGGCGTGTTTTGCTTAGGGCCGTG
Pr12091	GCGTCGACCTAATGATGATGATGGTGATGCAGCTCTTTCCAGTCGATCG
Pr12092	CATGCCATGGGCCTGCTGGCTACCTTCAAACTGT
Pr12093	GCGTCGACCTAATGATGATGATGGTGATGACAACGCGCTTCAGATGCCG
Pr12705	CGGAATTCAGGAGGATTACATATGAATCC
Pr12706	GGGGTACCCTAATGATGATGATGGTGATG
Pr12712	CTGTGCCCGCGCCAGGTCCAGGTTGACCTTGCTACGCGCATTCCACGG
Pr12713	CCGTGGAATGCGCGTAGCAAGGTCAACCTGGACCTGGCGCGGGCACAG
Pr12714	CGGAATTCAGGAGGATTACATATGACGAT
Pr12715	CTGCGCCTGACGCAGGTCTTTTTTCTCGTTGCTGTACTTGCCCCAAGG
Pr12716	CCTTGGGGCAAGTACAGCAACGAGAAAAAAGACCTGCGTCAGGCGCAG
Pr12717	CACGTCATACATGCGCTCCACGGTGAAACGCTGAACACCGAGATAGTC
Pr12718	GACTATCTCGGTGTTCAGCGTTTCACCGTGGAGCGCATGTATGACGTG
Pr12719	TTCGTTATCCGCGCGACCATAGTCGAACACGGGCTTCCCCACGAAGTC

Pr12720	GACTTCGTGGGGAAGCCCGTGTTCGACTATGGTCGCGCGGATAACGAA
Pr12721	CTGTGCCCGCGCCAGGTCCAGGTTGACCTTGCTACGCGCATTCCACGG
Pr12722	CCGTGGAATGCGCGTAGCAAGGTCAACCTGGACCTGGCGCGGGCACAG
Pr12723	TTCGTTATCCGCGCGACCATAGTCGAACACGGGCTTCCCCACGAAGTC
Pr12724	GACTTCGTGGGGAAGCCCGTGTTCGACTATGGTCGCGCGGATAACGAA
Pr12725	CTGCGCCTGACGCAGGTCTTTTTTCTCGTTGCTGTACTTGCCCCAAGG
Pr12726	CCTTGGGGCAAGTACAGCAACGAGAAAAAAGACCTGCGTCAGGCGCAG
Pr12727	CACGTCATACATGCGCTCCACGGTGAAACGCTGAACACCGAGATAGTC
Pr12728	GACTATCTCGGTGTTCAGCGTTTCACCGTGGAGCGCATGTATGACGTG
Pr13595	CATGCCATGGGCGCAGAACCGCGTCAGGAATTCGAA
Pr13596	GCGTCGACCTAATGATGATGATGGTGATGCAGACCCTGTTTCGCCAGACTTGC
Pr13599	CTCGTGGCGTGAGAGGATGCGTTTACCGCGTCTTTCAGTG
Pr13600	CACTGAAAGACGCGGTAAACGCATCCTCTCACGCCACGAG
Pr13601	GCGTCGACCTAATGATGATGATGGTGATG
Pr13615	TCCGGCAGACCGCCCATGTCGTTTACCGCGTCTTTCAGTG
Pr13616	CACTGAAAGACGCGGTAAACGACATGGGCGGTCTGCCGGA
Pr13617	GCGTCGACCTAATGATGATGATGGTGATGAGCAATGTGTTCGTGGTCAGCATC
Pr13620	GCGTCGACCTAATGATGATGATGGTGATGGTTTGCTTCTTTGATAGCAATGTG
Pr13623	GCGTCGACCTAATGATGATGATGGTGATGCGGAATACCCAGGTTGTTTGCTTC
Pr13731	GCGCGGTCTCACCAGGTTTACCGCGTCTTTCAGTGCTTT
Pr13732	GCGCGGTCTCACTGGGCGGTATCAAAGACATGGGC
Pr13733	GCGCGGTCTCAGTTTGTTTACCGCGTCTTTCAGTGCTTT
Pr13734	GCGCGGTCTCAAAACTGGAAAACAGCCTGGGCGGT

Table S3. The materials and conditions for production of the recombinant proteins

Gene ID[1]	Description	Abundance Ratio[2]	Primer set[3], plasmid[4]	Degradation[5]	Induction condition[6]
FTL528	Type III restriction enzyme	3.037	Pr10884/Pr10931, pST7529	No	37 ℃ 220 rpm 0.5 h
FTL1136	Hypothetical protein	2.691	Pr10900/Pr10933, pST7537	No	37 ℃ 220 rpm 2 h
FTL499	S-adenosylmethionine decarboxylase	2.347	Pr10882/Pr10883, pST7528	No	37 ℃ 220 rpm 0.5 h
FTL127	Formate dehydrogenase	2.243	Pr7757/Pr7758, pST5005	No	37 ℃ 220 rpm 2 h
FTL1218	PAS fold family protein	2.217	Pr7753/Pr7754, pST5010; Pr11207/Pr11208, pST7984	Yes	37 ℃ 220 rpm 1 h
FTL1917	30S ribosomal protein S6	2.060	Pr10912/Pr10913, pST7543	No	37 ℃ 220 rpm 2 h
FTL316	Arsenate reductase	1.966	Pr10878/Pr10930, pST7526; Pr11191/Pr11192, pST7976	Yes	37 ℃ 220 rpm 0.5 h
FTL1923	Zinc carboxypeptidase family protein	1.900	Pr10914/Pr10915, pST7544	No	37 ℃ 220 rpm 0.5 h
FTL196	UDP-3-O-[3-hydroxymy ristoyl] glucosamine N-acyltransferase	1.856	Pr10876/Pr10877, pST7525; Pr11189/Pr11190, pST7975	Yes	37 ℃ 220 rpm 0.5 h
FTL1244	Exodeoxyribonuclease III	1.842	Pr10928/Pr10929, pST7540	No	No expression
FTL160	LamB/YcsF family protein	1.789	Pr10874/Pr10875, pST7524	No	Inclusion body
FTL1566	CutC family protein	1.753	Pr10908/Pr10909, pST7541; Pr11213/Pr11214, pST7987	No	37 ℃ 220 rpm 0.5 h
FTL975	Nif3 family protein	1.661	Pr10892/Pr10893, pST7533	No	37 ℃ 220 rpm 2 h
FTL1003	DNA polymerase III subunit epsilon	1.652	Pr10896/Pr10897, pST7535; Pr11197/Pr11198, pST7979	Yes	37 ℃ 220 rpm 0.5 h
FTL1935	ABC transporter ATP-binding protein	1.632	Pr10916/Pr10917, pST7545; Pr11205/Pr11206, pST7983	Yes	16 ℃ 180 rpm 3 h
FTL965	ATP-dependent protease peptidase subunit	1.616	Pr10890/Pr10891, pST7532; Pr11209/Pr11210, pST7985	No	16 ℃ 180 rpm 3 h
FTL1644	Glycerol kinase	1.607	Pr10910/Pr10911, pST7542	No	37 ℃ 220 rpm 0.5 h
FTL995	Haloacid dehalogenase	1.591	Pr10894/Pr10895, pST7534; Pr11211/Pr11212, pST7986	No	37 ℃ 220 rpm 0.5 h
FTL455	Acetyltransferase	1.581	Pr10880/Pr10881, pST7527; Pr11193/Pr11194, pST7977	Yes	37 ℃ 220 rpm 0.5 h
FTL1034	Sulfate	1.570	Pr10898/Pr10932, pST7536;	Yes	16 ℃ 180

	adenylyltransferase		Pr11199/Pr11200, pST7980		rpm 3 h
FTL714	D-3-phosphoglycerate dehydrogenase	1.559	Pr10886/Pr10887, pST7530	No	Inclusion body
FTL1216	Hypothetical protein	1.531	Pr10904/Pr10905, pST7539; Pr11203/Pr11204, pST7982	Yes	37 ℃ 220 rpm 0.5 h
FTL1167	Hypothetical protein	1.499	Pr10902/Pr10903, pST7538; Pr11201/Pr11202, pST7981	Yes	37 ℃ 220 rpm 1 h
FTL964	ATP-dependent protease ATP-binding subunit HslU	1.493	Pr10888/Pr10889, pST7531; Pr11195/Pr11196, pST7978	Yes	37 ℃ 220 rpm 1 h
b169, *rpsB*	30S ribosomal subunit protein S2		Pr11811/Pr11812, pST8968	No	37 ℃ 220 rpm 0.5 h
b440, *hupB*	DNA-binding protein HUβ		Pr11813/Pr11814, pST8969	Yes	37 ℃ 220 rpm 0.5 h
b958, *sulA*	SOS cell division inhibitor		Pr11219/Pr11220, pST7990	Yes	37 ℃ 220 rpm 0.5 h
b1951, *rcsA*	Positive regulator of capsule biosynthesis		Pr11221/Pr11222, pST7991	Yes	37 ℃ 220 rpm 0.5 h
b3687, *ibpA*	Heat shock chaperone		Pr11215/Pr11216, pST7988	No	37 ℃ 220 rpm 0.5 h
b4062, *soxS*	Regulator of superoxide response regulon		Pr11217/Pr11218, pST7989	Yes	37 ℃ 220 rpm 0.5 h
TFAM2, MG824985	Transcription factor A, mitochondrial isoform 2		Pr12088/Pr12089, pST9320	Yes	37 ℃ 220 rpm 0.5 h
UNG1, MG824986	Uracil-DNA glycosylase isoform 1		Pr12090/Pr12091, pST9321	Yes	37 ℃ 220 rpm 0.5 h
STAR, MG824987	Steroidogenic acute regulatory protein, mitochondrial		Pr12092/Pr12093, pST9322	Yes	37 ℃ 220 rpm 0.5 h
GAPDH, NM_001289745.1	Glyceraldehyde-3-phosphate dehydrogenase		Pr11626/Pr11627, pST8721	Yes	37 ℃ 220 rpm 0.5 h

[1]*Francisella* and *E. coli* genes are available under the GenBank accessions NC_007880 (LVS genome) and NC_000913 (MG1655 genome).

[2]Quantitative abundance ratio of the protein in the Δ*lon* mutant vs. LVS.

[3]Primer set used to amplify the target genes.

[4]pACYCDuet and pEDL17 derivatives containing the target genes.

[5]Degradable or undegradable by fLon in *E. coli*.

[6]Induction condition for each target gene.

Table S4. The biochemical characteristics of *Francisella* Lon substrates

Gene	Description	Length[1]	Nonpolar AAs composition[2]	Aromatic AAs composition[3]	Cellular localization[4]
FTL196, *lpxD*	UDP-3-O-[3-hydroxymyristoyl] glucosamine N-acyltransferase	347	41.6	5.9	Cytoplasm
FTL316	Arsenate reductase	117	37.7	12.9	Cytoplasm
FTL455	Acetyltransferase	171	34.6	18.7	Cytoplasm
FTL578, *ocd*	Ornithine cyclodeaminase	282	42.5	7.0	Cytoplasm
FTL663	Heat shock protein	122	34.3	7.3	Cytoplasm
FTL964, *hslU*	ATP-dependent protease ATP-binding subunit HslU	455	42.2	3.9	Cytoplasm
FTL1003, *dnaQ*	DNA polymerase III subunit epsilon	238	42	9.3	Cytoplasm
FTL1034, *cysN*	Sulfate adenylyltransferase	317	38.8	7.6	Cytoplasm
FTL1167	Hypothetical protein	173	45.2	5.3	Cytoplasm
FTL1216	Hypothetical protein	194	40.7	8.7	Cytoplasm
FTL1217	Heat shock protein	123	35.8	7.6	Cytoplasm
FTL1218	PAS fold family protein	329	39.8	10.4	Cytoplasm
FTL1228, *sufD*	SufS activator complex, SufD subunit	381	41.1	9.1	Cytoplasm
FTL1935	ABC transporter ATP-binding protein	225	40.9	8.9	Cytoplasm
FTL1957	Heat shock protein	142	31.6	9.1	Cytoplasm

[1]The total amino acids number of each protein.

[2]Percentage of the nonpolar amino acids (Ala, Val, Leu, Ile, Pro, Phe, Trp, and Met) in each protein.

[3]Percentage of the aromatic amino acids (Tyr, Phe, and Trp) in each protein.

[4]Subcellular localization of each protein is predicted based on signal peptide prediction with web-based SignalP 4.1 (http://www.cbs.dtu.dk/services/SignalP/) and transmembrane helices prediction with TMHMM 2.0 ((http://www.cbs.dtu.dk/services/TMHMM/).

REFERENCES

1 Su, J. *et al.* Genome-wide identification of *Francisella tularensis* virulence determinants. *Infect Immun* **75**, 3089-3101 (2007).

2 Guzman, L. M., Belin, D., Carson, M. J. & Beckwith, J. Tight regulation, modulation, and high-level expression by vectors containing the arabinose PBAD promoter. *J Bacteriol* **177**, 4121-4130 (1995).

3 LoVullo, E. D., Miller, C. N., Pavelka, M. S., Jr. & Kawula, T. H. TetR-based gene regulation systems for *Francisella tularensis*. *Appl Environ Microbiol* **78**, 6883-6889 (2012).

4 He, L. *et al.* The protease locus of *Francisella tularensis* LVS is required for stress tolerance and infection in the mammalian host. *Infect Immun* **84**, 1387-1402 (2016).
